# SARS-CoV-2 modulates virus receptor expression in placenta and can induce trophoblast fusion, inflammation and endothelial permeability

**DOI:** 10.3389/fimmu.2022.957224

**Published:** 2022-09-13

**Authors:** Chiara Agostinis, Miriam Toffoli, Mariagiulia Spazzapan, Andrea Balduit, Gabriella Zito, Alessandro Mangogna, Luisa Zupin, Tiziana Salviato, Serena Maiocchi, Federico Romano, Sergio Crovella, Francesco Fontana, Luca Braga, Marco Confalonieri, Giuseppe Ricci, Uday Kishore, Roberta Bulla

**Affiliations:** ^1^ Institute for Maternal and Child Health, Istituto di Ricovero e Cura a Carattere Scientifico (IRCCS) Burlo Garofolo, Trieste, Italy; ^2^ Department of Life Sciences, University of Trieste, Trieste, Italy; ^3^ Department of Diagnostic, Clinic and Public Health Medicine, University of Modena and Reggio Emilia, Modena, Italy; ^4^ Functional Cell Biology Group, International Centre for Genetic Engineering and Biotechnology (ICGEB), Trieste, Italy; ^5^ Biological Science Program, Department of Biological and Environmental Sciences, College of Arts and Sciences, Qatar University, Doha, Qatar; ^6^ Division of Laboratory Medicine, Azienda Sanitaria Universitaria Giuliano Isontina (ASU GI), Trieste, Italy; ^7^ Department of Medical, Surgical and Health Science, University of Trieste, Trieste, Italy; ^8^ Biosciences, College of Health, Medicine and Life Sciences, Brunel University of London, London, United Kingdom; ^9^ Department of Veterinary Medicine, United Arab Emirates University, Al Ain, United Arab Emirates

**Keywords:** SARS-CoV-2, ACE2, TMPRSS2, CD147, pregnancy

## Abstract

SARS-CoV-2 is a devastating virus that induces a range of immunopathological mechanisms including cytokine storm, apoptosis, inflammation and complement and coagulation pathway hyperactivation. However, how the infection impacts pregnant mothers is still being worked out due to evidence of vertical transmission of the SARS-CoV-2, and higher incidence of pre-eclampsia, preterm birth, caesarian section, and fetal mortality. In this study, we assessed the levels of the three main receptors of SARS-CoV-2 (ACE2, TMPRSS2 and CD147) in placentae derived from SARS-CoV-2 positive and negative mothers. Moreover, we measured the effects of Spike protein on placental cell lines, in addition to their susceptibility to infection. SARS-CoV-2 negative placentae showed elevated levels of CD147 and considerably low amount of TMPRSS2, making them non-permissive to infection. SARS-CoV-2 presence upregulated TMPRSS2 expression in syncytiotrophoblast and cytotrophoblast cells, thereby rendering them amenable to infection. The non-permissiveness of placental cells can be due to their less fusogenicity due to infection. We also found that Spike protein was capable of inducing pro-inflammatory cytokine production, syncytiotrophoblast apoptosis and increased vascular permeability. These events can elicit pre-eclampsia-like syndrome that marks a high percentage of pregnancies when mothers are infected with SARS-CoV-2. Our study raises important points relevant to SARS-CoV-2 mediated adverse pregnancy outcomes.

## Introduction

COVID-19 pandemic has triggered an explosion of research focusing on host-pathogen interaction mostly due to the urgency of developing, administering and monitoring a range of vaccine candidates (1, 2). While the mode of infection of target cells by SARS-CoV-2 and its modulation of innate and adaptive immune system, together with coagulation pathway, is beginning to unravel, many aspects of the interplay between SARS-CoV-2 and immune system during pregnancy remain unclear. The impact of COVID-19 on pregnant women’s health and fetal development is now being recognised, but the mechanisms and the evidence for vertical transmission of the virus from the mother to the fetus are not well established. No consensus has been reached yet about the percentage of vertical transmission, with recent findings reporting 2-3% of cases (3, 4). Moreover, the risk of pre-term delivery, pre-eclampsia (PE), cesarean section, severe neonatal/perinatal morbidity and mortality has been found to be significantly increased in COVID-19 pregnant women (5–9). COVID-19 patients show a higher incidence of PE (8,1%), as compared with the non-infected mothers (4,4%) (10, 11), independent of preexisting conditions and other risk factors, such as obesity, diabetes and hypertension (12). Similar pathophysiological association has also emerged between COVID-19 and PE, due to endothelial injury, which leads to an overall inflammatory microenvironment, characterized by an increase in serum and placental levels of pro-inflammatory and decrease in anti-inflammatory cytokines, and the pivotal involvement of complement system (13–15).

The infection of host cells by SARS-CoV-2 requires the presence of transmembrane receptor Angiotensin-Converting Enzyme 2 (ACE2) and Type II Transmembrane Serine Protease (TMPRSS2) (16, 17). SARS-CoV-2 may bind to a third alternative receptor, the cluster of differentiation 147 (CD147)/Extracellular matrix metalloproteinase inducer (EMMPRIN)/Basigin (BSG) (18). Among the different receptors for the entry of SARS-CoV-2 that were discovered later, CD147 is one of the key surface molecules involved in regulating implantation, invasion and differentiation of human trophoblasts (19). CD147 is a widely expressed membrane protein of the immunoglobulin superfamily that has been implicated in tissue remodeling and in pathological conditions such as atherosclerosis, aneurysm, heart failure, osteoarthritis, and cancer (20). Furthermore, it is involved in HIV-1 infection by interacting with virus-associated cyclophilin A (21), and in other viral infections, especially SARS-CoV-2 infection. CD147 mediates macrophage activation that results in the expression of matrix metallopeptidase-9, and pro-inflammatory cytokines and chemokines in endothelial cells (22, 23).

SARS-CoV-2 entry receptors, such as CD147 and ACE2, are thought to allow the virus to enter the cell cytoplasm and activate the NLRP3 inflammasome, resulting in the cleavage of interleukin (IL)-1β and IL-18 cytokines (18). SARS-CoV-2 binds ACE2, then TMPRSS2 primes the SARS-CoV-2 Spike protein which subsequently allows the membrane fusion and the entry into the host cell (16, 24). Immunohistochemical analyses in placentae exposed to maternal SARS-CoV-2 demonstrated strong expression of ACE2 in syncytiotrophoblast and cytotrophoblast across gestation, but a weak expression of TMPRSS2, most often confined to the fetal endothelium or unquantifiable by conventional immunohistochemical methods (25). This differential expression of ACE2 and TMPRSS2 is likely responsible for the ongoing debate on the vertical transmission of SARS-CoV-2. Indeed, some placentae lack colocalization of ACE2 and TMPRSS2 (26–28). Another important aspect rendering pregnant women more susceptible to severe COVID-19 is the capacity of SARS-CoV-2 to reduce ACE2 expression in placenta (7). Since ACE2 plays an important role in the regulation of maternal hemodynamics and the development of the placenta (29, 30), its reduced level can become a risk factor for both mother and fetus, resulting in impaired placental vascularity, increased maternal blood pressure, and adverse pregnancy outcomes with placental dysfunction, which can also aggravate pre-existing conditions in pregnant women, such as chronic hypertensive disorders, chronic renal disease, diabetes and pulmonary comorbidities (30–33).

There is therefore a lack of information on how maternal infection with SARS-CoV-2 impacts the expression of ACE2 and TMPRSS2 in the placenta. Here, we compared term placentae collected before the COVID-19 pandemic and SARS-CoV-2+ placentae, analyzing the expression of ACE2, TMPRSS2 and CD147. Receptor expression was investigated also in isolated placental cells and cell lines by Real-Time (RT)-qPCR, Western blot and flow cytometry. Finally, we evaluated the effects of cell stimulation with Spike protein and SARS-CoV-2 capability to infect placental cells *in vitro*.

## Materials and methods

### Reagents and antibodies

The following antibodies were used: rabbit polyclonal anti-ACE2 (#SAB2100025), anti-mouse polyclonal Immunoglobulins (IgG, IgA, IgM) alkaline phosphatase (AP)-conjugate and anti-rabbit IgG AP-conjugate were purchased from Sigma-Aldrich (St. Louis, MO, USA); rabbit polyclonal anti-TMPRSS2 (#PA5-116054), rabbit monoclonal anti-CD147 (#MA5-29060), anti-mouse IgG Alexa Fluor^®^ 488-conjugate, Alexa Fluor^®^ 568 phalloidin (#A12380), and V5 Tag Monoclonal Antibody Alexa Fluor 488-conjugate (#37-7500-A488) were bought from Invitrogen; SARS-CoV/SARS-CoV-2 (COVID-19) spike antibody (#GTX632604) was purchased from GeneTex, while anti-SARS/SARS-CoV-2 Coronavirus Nucleocapsid Antibody (#MA1-7403) was from Thermo Fisher Scientific. SARS-CoV-2 Spike protein S1 (aa 1-674) Recombinant antigen was bought from Merck Millipore (Darmstadt, Germania). All other chemicals were purchased from Sigma-Aldrich.

### Tissue samples

Term placental tissues were obtained from 7 control healthy women and 8 SARS-CoV-2+ women after delivery. Patients’ characteristics are summarised in **Table 1**.

**Table 1:** Clinical characteristics of women enrolled in the study.

Characteristics	SARS-CoV-2+ (n=8)	Healthy controls (n=7)
Mean maternal age, years (SD)	34 (3.8)	35 (0.7)
Gestational age at delivery, weeks (SD)	36.4 (4.6)	37.6 (3.8)
Mode of delivery		
Vaginal delivery, % (n)	0% (0)	71.4% (5)
Cesarean section, % (n)	100% (8)	28.6% (2)
Pregnancy complications		
Pre-eclampsia, % (n)	0% (0)	0% (0)
COVID-19 manifestations		
Asymptomatic, % (n)	25% (2)	n.a.
Paucisymptomatic, % (n)	75% (6)	n.a.
- Fever <37.5°C, % (n)	100% (6)	n.a.
- Cough, % (n)	100% (6)	n.a.
- Loss of smell and taste, % (n)	66.6% (4)	n.a.

SD, standard deviation; n, number; n.a., not applicable.

Placental and decidual biopsy specimens were obtained from women undergoing elective termination of pregnancy at 8-12 weeks of gestation, or from term subjects undergoing caesarean delivery.

The study was approved by the institutional review board of The Maternal-Children’s Hospital (RC 40/20, IRCCS “Burlo Garofolo,” Trieste, Italy), and informed consent was obtained from the participating patients.

### Cell lines

JAR and BeWo cells were purchased from American Type Culture Collection (ATCC). HTR-8/SVneo was kindly donated by Prof. P.K. Lala (University of Western Ontario, Canada). This cell line was derived from placental villi of the first trimester immortalized by pSV3-neo containing the regions of the SV40 coding for the SV3 Tag. All the placental cell lines were cultured in RPMI 1640 + Glutamax Medium supplemented with 1% v/v penicillin–streptomycin (PS, Sigma-Aldrich) and 10% v/v fetal bovine serum (FBS, Life Technologies). For JAR cell line, a coating of 50 μg/mL fibronectin (Sacco) was needed. Vero E6 cell line (ATCC CRL-1586) was grown in MEM + 10% FBS.

### Cell isolation and culture

Human umbilical vein endothelial cells (HUVECs) were isolated from umbilical cords of healthy placentae following the protocol by Jaffe rom (34) and cultured with Human Endothelial Serum-Free Medium (HESFM, Life Technologies, Carlsbad, CA, USA) supplemented with 20 ng/mL of epidermal growth factor (EGF), 10 ng/mL of basic fibroblast growth factor (bFGF) (Immunological Sciences), 1% PS (Sigma-Aldrich) and 10% FBS (Life Technologies). Decidual stromal cells (DSCs) and Decidual endothelial cells (DECs) were isolated from first trimester decidual biopsy specimens as previously described (35). Cells were respectively cultured in RPMI 1640 + Glutamax Medium supplemented with 1% PS and 10% FBS, and HESFM supplemented with 20 ng/mL of EGF, 10 ng/mL of bFGF (Immunological Sciences), 1% PS and 10% FBS. For DECs, a coating with 50 μg/mL fibronectin was needed. Extravillous trophoblasts (EVTs) were purified from placental tissue incubated in Hanks’ balanced salt solution (HBSS) containing 0.25% trypsin and 0.2 mg/mL DNase I (Roche) for 20 min at 37°C. Cells were seeded on 25-cm^2^ flask pre-coated with 5 μg/cm^2^ fibronectin and cultured with RPMI 1640 +Glutamax Medium supplemented with 1% PS and 10% FBS, and used within 12 h.

### Immunohistochemical analysis

Term Placental tissues from SARS-CoV-2+ women and control tissues from non-infected healthy mothers were fixed in 10% buffered formalin, paraffin-embedded and stored at 4°C. Sections were deparaffinized with xylene and rehydrated with decreasing concentrations of ethanol (100%, 95%, 70%) and dH_2_O. To perform antigen retrieval, sections were kept for 20 min at 95°C in Citrate buffer, pH 6, or in Tris-HCl/EDTA buffer, pH 9. Neutralization of the endogenous peroxidase was performed adding H_2_O_2_ for 5 min, and then, the sections were incubated with PBS + 2% BSA for 30 min to block non-specific binding. Next, the following antibodies were incubated overnight at 4°C: anti-ACE2 (1:200), anti-TMPRSS2 (1:50), anti-CD147 (1:2500) and anti-SARS-CoV--2 spike (1:200) antibodies. Anti-rabbit horseradish peroxidase (HRP)-conjugate (1:500) and anti-mouse HRP-conjugate (1:250) were than incubated for 30 min at RT, prior to detection using AEC kit (Vector Laboratories). Sections were counterstained with Mayer's haematoxylin (DiaPath) and examined under a Leica DM 2000 optical microscope. Images were collected using a Leica DFC 7000 T digital camera (Leica Microsystems, Wetzlar, Germany).

To quantify the expression of entry receptors in placentae, we utilized an immunoreactive score (IRS), which is commonly used for immunohistochemical evaluation (36). For each slide, we analyzed three different visual fields of the microscope, attributing a score of positivity ranging from 0 to 4.

### Real time quantitative PCR

Cells (EVT, DEC, DSC, JAR, BeWo, HTR8 or HUVEC) were grown into a 24-well plate under resting conditions, and/or stimulated with SARS-CoV-2 Spike S1 recombinant protein (10 ng/mL and 1000 ng/mL). Total RNA was isolated using a RNA purification kit (Norgen Biotek company, Thorold, ON, Canada). The isolation of RNA from tissue samples was carried out using Phenol-Chloroform extraction method, and RNA quantification was done *via* NanoDrop^®^ spectrometer (NanoDrop). RNA was converted to cDNA using SensiFAST™ cDNA Synthesis Kit (Meridian Bioscience, Memphis, TN, USA). The expression level of the following genes was evaluated: ACE2, TMPRSS2, CD147, IL-6, IL-8 and TNF-α. The sequences of the primers used are included in **Table 2**. The reaction was performed by the Rotor-Gene 6000 (Corbett, Explera), *via* 45 cycles of denaturation (60 sec at 95°C), annealing (30 sec at 60°C, melting temperature of the primers) and amplification (60 sec at 72°C). The expression level of the different genes analysed was then evaluated *via* a comparative quantification using Rotor Gene 1.7 Software (Corbett Research) and normalized with the expression of the housekeeping genes (18S, GAPDH and ACTB) (37). To evaluate the viral replication in cells, the CDC primers and probe for N gene (Eurofins, Luxembourg) and the Luna^®^ Universal Probe One-Step RT-qPCR Kit (New England Biolabs, Ipswich, MA, USA) were used *via* the 7500 Fast Real-Time PCR system (Thermo Fisher Scientific, Waltham, MA, USA) following the cycle of 50°C for 10 min, 95°C for 1 min, and then 40 cycles at 95°C for 10 min, 60°C for 30 min. Standard curve for quantification was generated using the nCoV-CDC-Control Plasmid (Eurofins).

**Table 2:** Primer sequences used for RT-qPCR.

Protein	Gene	Sense	Gene Sequence (5’ → 3’)	Tm (°C)	NCBI GeneID
Angiotensin Converting Enzyme 2	ACE2	Forward Reverse	ACAGTCCACACTTGCCCAAAT TGAGAGCACTGAAGACCCATT	60	NM_001371415.1
Transmembrane Serine Protease 2	TMPRSS2	Forward Reverse	GAT TAG CCG TCT GCC CTC ATT TGT CAG GAG TGT ACG GGA ATG TGA TGG	58	NM_001135099.1
BSG basigin	CD147	Forward Reverse	CTA CAC ATT GAG AAC CTG AAC TTC TCG TAG ATG AAG ATG ATG	60	NM_001728.4
Interleukin 6	IL-6	Forward Reverse	GTA CAT CCT CGA CGG CAT C CCA GGC AAG TCT CCT CAT TG	60	NM_000600.3
C-X-C motif chemokine ligand 8	IL8/CXCL8	Forward Reverse	AGG TGC AGT AGT TTT GCC AAG GA TTT CTG TGT TGG CGC AGT GT	60	NM_000584
Tumor Necrosis Factor-α	TNF	Forward Reverse	GGC CCA GGC AGT CAG ATC AT GGG GCT CTT GAT GGC AGA GA	65	NM_000594.3
C-C motif chemokine ligand 2	MCP1/CCL2	Forward Reverse	ATC AAT GCC CCA GTC ACC AGT CTT CGG AGT TTG GG	60	NM_002982.3
CCL5 C-C motif chemokine ligand 5	RANTES/CCL5	Forward Reverse	TAC CAT GAA GGT CTC CGC GAC AAA GAC GAC TGC TGG	60	NM_002985.3
C-X-C motif chemokine ligand 10	IP10/CXCL10	Forward Reverse	AGG AAC CTC CAG TCT CAG CA CAA AAT TGG CTT GCA GGA AT	60	NM_001565.4
Ribosomal protein S18	RPS18	Forward Reverse	ATC CCT GAA AAG TTC CAG CA CCC TGT TGG TGA GGT CAA TG	60	NM_022551.2
Actin Beta	ACTB	Forward Reverse	CTA CAA TGA GCT CCG TGT GG AAG GAA GGC TGG AAG AGT GC	60	NM_001101.3
Glyceraldehyde-3-phosphate dehydrogenase	GAPDH	Forward Reverse	GAT CAT CAG CAA TGC CTC CT GT GGT CAT GAG TCC TTC CA	60	NM_002046.5
SARS-CoV-2 Nucleocapsid Protein	2019-nCoV_N2	Forward Reverse Probe	TTA CAA ACA TTG GCC GCA AA GCG CGA CAT TCC GAA GAA FAM-ACA ATT TGC CCC CAG CGC TTC AG-BHQ1		43740575

### Western blot analysis

To assess the protein level of ACE2, TMPRSS2 and CD147, 1.2 x 10^6^ cells (JAR, BeWo, HTR8 or HUVEC) were lysed. Proteins were than separated *via* 10% SDS-PAGE under reducing conditions and transferred to a nitrocellulose membrane using the semi-dry Trans-blot Turbo Transfer System (BIORAD). A blocking step with 5% skimmed milk in Tris-Buffered Saline + Tween 20 (TBST, 10 mM Tris, pH 8.0, 150 mM NaCl, 0.5% Tween 20) was followed by an overnight incubation at 4°C with primary antibody anti-ACE2 (1:1000), anti-TMPRSS2 (1:1000) and anti-CD147(1:1000). After washing the membrane three times in TBST, the blot was incubated with anti-rabbit LI-COR IRDye 800CW (1:10000) for 1 h at RT. Another washing step was performed, and the fluorescence intensity was acquired using Odyssey^®^CLx near-infrared scanner (LI-COR Biosciences, Lincoln, NE, USA) and processed with Image Studio Ver 5.2 (LI-COR Bioscience). Data were normalized for the signal of β-Actin.

### Flow cytometry

4 x 10^5^ cells (JAR, BeWo, HTR8 or HUVEC) were incubated with primary antibodies- anti-ACE2 (1:100), anti-TMPRSS2 (1:100), anti-CD147 (1:100) diluted in dPBS + 2% BSA, 0.7 mM CaCl_2_ and 0.7 mM MgCl_2_ for 45 min on ice. Incubation with anti-rabbit FITC-conjugate was performed for 30 min on ice in the dark. The cells were than resuspended and fixed in 1% paraformaldehyde (PFA). The same number of cells were also analyzed following an initial fixation step with 3% PFA in the dark for 20 min. Fluorescence was acquired with the FACScalibur (BD Bioscience, San Jose, CA, USA), and the data was processed using FlowJo™ v 10.7.2 Software (BD Life Sciences).

### Evaluation of apoptosis

To evaluate the level of apoptosis induced by SARS-CoV-2, the CellEvent Caspase 3/7 Detection Reagent (ThermoFisher) was used. Cells (JAR, BeWo, HTR8 or HUVEC) were seeded in a 96 well-plate and allowed to reach 80% confluence. Cells were then stimulated with recombinant SARS-CoV-2 Spike S1 protein (10 ng/mL or 1000 ng/mL); H_2_O_2_ was used as a positive control. After 6 h, 5 μM CellEvent reagent was added to the wells and incubated for 30 min. The CellEvent reagent is four-amino acid peptide (DEVD)-conjugated to a nucleic acid-binding dye: activated caspases 3/7 cleave the DEVD peptide allowing the dye to bind to DNA and produce a fluorescent emission (∼530 nm). Fluorescence intensity was than measured by an INFINITE200 (TECAN).

### Measurement of receptor expression on the membrane

Placental cells (JAR, BeWo, HTR8 or HUVEC) were cultured in a 96 well-plate (Corning) and allowed to reach 100% of confluence to perform an ELISA on the whole cells. Cells were stimulated overnight with SARS-CoV-2 Spike Protein S1 (10 ng/mL or 1000 ng/mL). Next day, cells were incubated with primary antibodies against SARS-CoV-2 receptors diluted in PBS + 2% BSA + 0.7 mM CaCl_2_ + 0.7 mM MgCl_2_ for 90 min at RT. Secondary antibody AP-conjugate were then incubated for 1h at RT. pNPP (ChemCruz) solution was added and the absorbance was read at 405 nm using a plate reader (PowerWaveX, Bio-Tek Instruments).

### Evaluation of placental cell susceptibility to SARS-CoV-2 infection *in vitro*

Infection experiments were carried out in the BSL-3 laboratory of San Polo Hospital ASUGI (Monfalcone, GO, Italy). SARS-CoV-2 virus, derived from nasopharyngeal swab (beta and delta variant), expanded and isolated from Vero E6 cells, was employed in the experiment. Vero E6 cells, which are highly permissive to SARS-CoV-2 replication, were used as a positive control.

Vero E6, HUVECs, HTR-8/SVneo, JAR and BeWo cells were seeded in a 24 well-plate a day prior to setting up infection assay in order to obtain a 70-80% of confluence. SARS-CoV-2 was diluted in serum-free medium and added to the cells at a multiplicity of infection (MOI) of 0.4. After 1 h incubation at 37°C, the medium was removed, cells were washed with PBS and fresh medium was added. Vero E6 cells were maintained in MEM supplemented with 2% FBS; BeWo, JAR and HTR-8/SVneo were cultured in RPMI + 2% FBS, while HUVECs was grown in serum-free HESFM. The cells were monitored every day for 7 days using an optical microscope for any cytopathic effect induced by viral infection. To evaluate the viral replication, the viral RNA load was assessed in the supernatant at different time points and intracellularly at the 7^th^ day post-infection. Briefly, at day 0, 4 and 7, 15 μL of supernatant were mixed with 45 μL of distilled water and subjected to thermolysis for RNA extraction (98°C for 3 min and 4°C for 5 min). The viral load was then quantified by RT-qPCR.

To assess if pre-treatment of the cells can improve the susceptibility towards the infection, cells were challenged with SARS-CoV-2 for 24 h (MOI=0.4). After washing, cells were challenged with the virus (MOI=0.4) for 1 h and the viral load was measured at day 1 and at day 4 post infection, as described above.

### Cell transfection with delta spike and cell fusion assays

BeWo, JAR, HTR-8/SVneo and HUVEC cells were incubated for 24 h with SARS-CoV-2 (MOI=0.4). Next, cells were washed in PBS, lysed, and RNA was isolated by the total RNA purification kit (Norgen Biotek company, Thorold, ON, Canada). JAR and HTR-8/SVneo cells were seeded at 1.2 x 10^4^ cells/well in a 96-well plate (CellCarrierUltra, Optical Plate, PerkinElmer). After 10 h, cells were transfected with: (a) pCMV-SPIKEDelta-V5 + PCMV-hACE2; (b) pCMV-SPIKEDelta-V5 + pcDNA3; (c) pCMV-GFP + pcDNA3. For each condition, a transfection mix was prepared with OPTIMEM (Gibco #31985062), FuGENE (Promega, #E2311) and both plasmids at the ratio of 50 μl OPTIMEM: 3,5 μl FuGENE: 1 μg total pDNA. Plasmids in each mix were added at 1:1 ratio. Each transfection mix was incubated at RT for 15 min, then 10 μl of mix was added to each well (200 ng of total DNA per well). The plate was incubated at 37°C under 5% CO_2_ for 24 h.

### Immunofluorescence microscopy

Cells (JAR, BeWo, HTR8 or HUVEC) were seeded onto round glass coverslip of 10 mm diameter and allowed to reach up to 70% confluence. Cells were then fixed with 3% PFA for 15 min in the dark. Blocking, permeabilization and quenching were sequentially carried out using 1% BSA, 0.1% Triton X-100 and 50 mM glycine in PBS, respectively, for 30 min at RT. Primary antibodies diluted in PBS + 2% BSA were added for 1 h, followed by incubation with anti-rabbit Cy3-conjugate (1:300) for 30 min at RT. Nuclei were stained with DAPI. Glass slides were mounted using Fluorescence Mounting Medium (Dako) and images were acquired with various devices as described in the figure legends.

To assess the viral entry at different time points and the transfection after 12 h, cells were fixed and permeabilized as described above. First, the non-specific binding of the antibody was blocked by 1 h of incubation with PBS-T + 10% normal goat serum (NGS). Cells were incubated overnight with anti-SARS/SARS-CoV-2 Coronavirus Nucleocapsid Antibody (1:100 in PBS-T + 1.5% NGS), and then with the anti-mouse Alexa Fluor 488-conjugate (1:500) for 1 h coupled with Alexa Fluor 569-conjugated Phalloidin (1:400). Subsequently, the coverslips were sealed on glass slides with a DAPI mounting medium (FluoroshieldTM with DAPI, Sigma Aldrich, Merck KGaA, Darmstadt, Germany). To evaluate the transfection with Delta Spike, cells were blocked with PBS + 1% BSA for 30 min. The staining for the Spike protein was then performed with anti-V5 Alexa 488 antibody (1:500 in BSA) for 2 h at RT and nuclei were stained with HOECHST 33342 (1:5000 in PBS, Invitrogen). A total of 15 fields per well were acquired and analyzed for the formation of cellular syncytia using the Harmony software (PerkinElmer) (38). Three independent biological replicates were run.

### Permeability assay

HUVECs (2 x 10^4^ cells) were seeded on to a fibronectin-coated (20 μg/mL) polycarbonate insert in a 24-well transwell (TW) system (6.5 mm diameter, 8-μm pores; Corning Costar, Milan, Italy) and incubated for 4 days, to allow the cells to form a functional monolayer. TWs were then tested for the formation of an intact endothelial monolayer by adding 1 mg/mL FITC-BSA (Sigma-Aldrich) in the upper chamber; the amount of labelled BSA that leaked through to the lower TW chamber was measured by an INFINITE200 (TECAN). When the fluorescence intensity was negligible, the following stimuli were added to the upper chamber: SARS-CoV-2 Spike Protein S1 (Sigma-Aldrich) (10 ng/mL or 1000 ng/mL) or 0.1 μg/mL Histamine (Sigma-Aldrich) as a positive control. The leaking of FITC-BSA was evaluated after 15 and 30 min.

### Statistical analysis

Mean and standard deviations were calculated for continuous variables, whereas frequencies and percentages were reported for categorical variables. Non-parametric data were assessed by Mann-Whitney U-tests. Patient data were analyzed by T-Student test. Analysis of different groups of data was performed using two-way analysis of variance (ANOVA). Results were expressed as mean ± standard deviations and p-values <0.05 were considered statistically significant. All statistical analyses were performed using GraphPad Prism software 9.0 (GraphPad Software Inc., La Jolla, CA, USA).

## Results

### Expression and distribution of ACE2, TMPRSS2 and CD147 in healthy placenta

**Figure 1:**
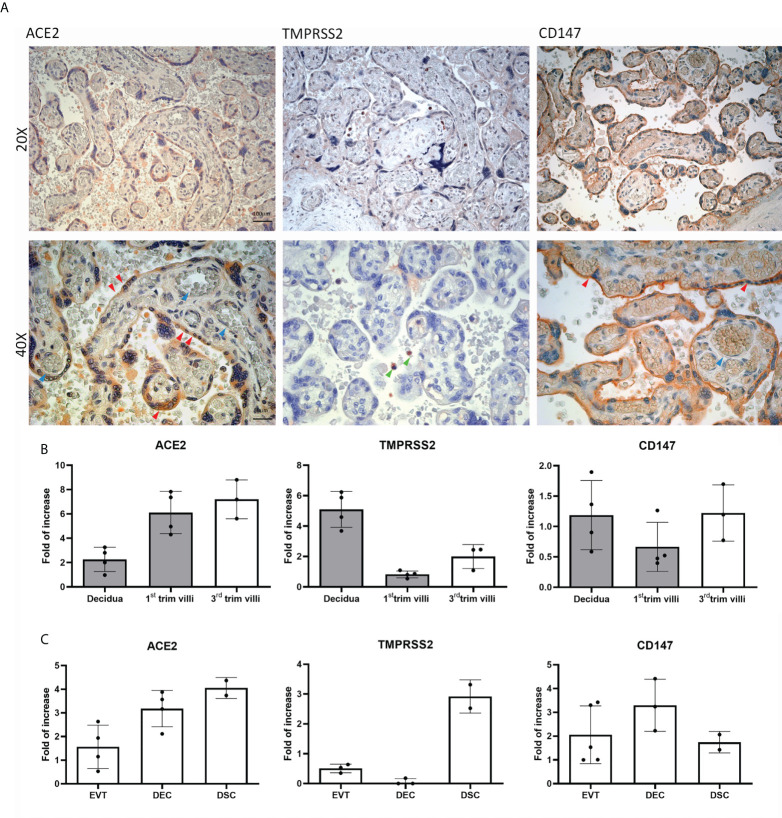
Distribution and basal expression of the SARS-CoV-2 receptors in healthy placentae. **(A)** Representative microphotographs showing ACE2, TMPRSS2 and CD147 distribution in placental tissues. ACE2 was localized within syncytiotrophoblasts, whilst TMPRSS2 staining was almost negative. Staining for CD147 indicated a high protein expression. AEC chromogen (red) was used to visualize the binding of anti-human primary antibodies. Red arrows indicate syncytiotrophoblast, blue arrows villous vessels and green arrows show leukocytes in the intervillous space. Nuclei were counterstained in blue with Mayer's Hematoxylin; scale bars, 100 µm. **(B)** Analysis of mRNA expression of SARS-CoV-2 receptors in placental tissues derived from healthy placentae: 5 from first trimester (grey histograms) and 3 from third trimester placentae. Data are represented as mean ± SE of three independent experiments performed in triplicate. **(C)** Histograms representing *ACE2*, *TMPRSS2* and *CD147* mRNA expression in extravillous trophoblast (EVT), endothelial (DEC) and stromal (DSC) cells isolated from normal first trimester decidua. Data are represented as mean ± SE of three independent experiments performed in triplicate.

To elucidate the mechanism of SARS-CoV-2 infection of the placenta, we first evaluated the basal expression and distribution of the main SARS-CoV-2 receptors: ACE2, TMPRSS2 and CD147 (**Figure 1A**), *via* immunohistochemistry (IHC) using 5 healthy term placentae.

IHC analysis revealed ACE2 expression on the syncytiotrophoblast monolayer (red arrows), with highly variable intensity (**Supplementary Figures 1**, **2**). The staining for TMPRSS2 expression was either very weak or almost absent, with the exception of some isolated cells in the intervillous space, which are likely polymorphonuclear leukocytes (green arrows). CD147 staining was strong in healthy placental tissues, being mainly present in the brush border of syncytiotrophoblasts. We also evaluated the local gene expression of the receptors, performing RT-qPCR on first and third trimester placental tissues as well as isolated placental cells. As shown in **Figure 1B**, *ACE2* transcript was detected in all the tissue samples analyzed, with higher expression in third trimester placentae. The mRNA expression of *CD147* was particularly elevated in first trimester decidua, showing an increase from first to third trimester in placental villi. A similar pattern of increase from first to third trimester villi was observed for *TMPRSS2* mRNA. With respect to first trimester isolated placental cells, we observed high expression of ACE2 and CD147 transcripts (**Figure 1C**) in all cell populations analyzed: EVT, DECs and DSCs. On the contrary, mRNA levels of TMPRSS2 were elevated only in DSC population (**Figure 1C**).

In summary, in healthy conditions, we noticed a variable expression of ACE2 by trophoblast cells (especially syncytiotrophoblast), a very low presence of TMPRSS2, and a strong expression of CD147.

### ACE2, TMPRSS2 and CD147 expression in placental cell lines

**Figure 2:**
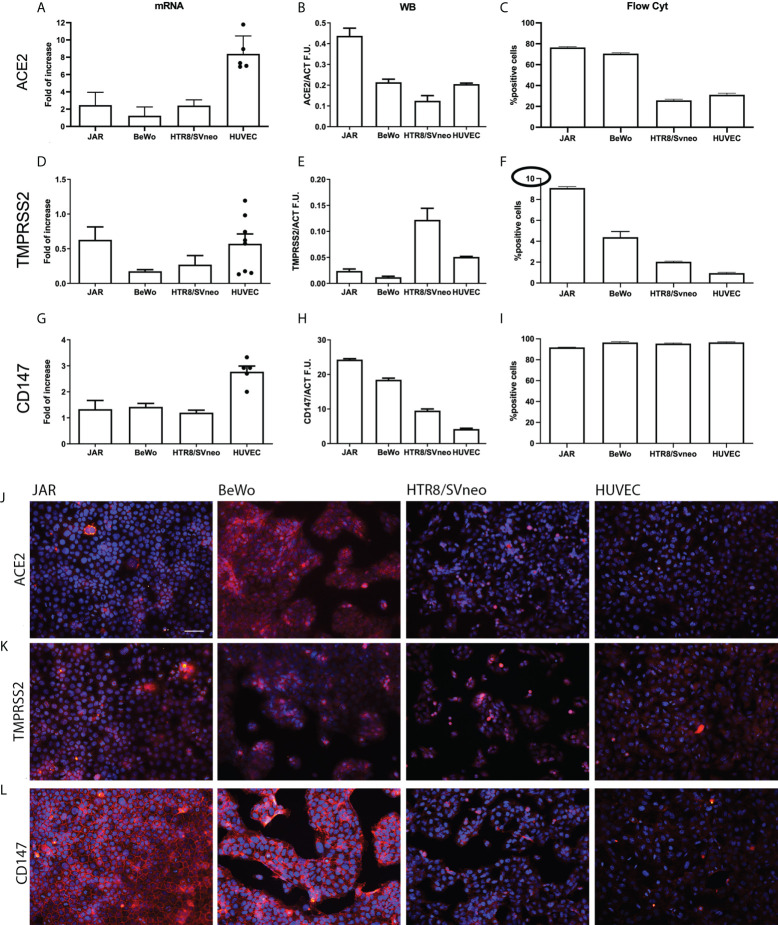
Characterization of SARS-CoV-2 entry receptors’ expression in placental cell lines. Gene and protein expression levels of ACE2 **(A-C)**, TMPRSS2 **(D-F)** and CD147 **(G-I)** were evaluated by RT-qPCR, Western blot and flow cytometry in JAR, BeWo, HTR8/SVneo and HUVECs (n = 6, different populations are represented by dots). All the placental cell lines showed a low level protein expression of TMPRSS2 (highlighted by the circle in the different set up of the axis) and a high protein expression level of CD147, whilst ACE2 expression appeared more variable among them. **(J-L)** Immunofluorescence analysis of SARS-CoV-2 entry receptors in placental cell lines. JAR, BeWo, HTR8/SVneo and HUVECs were stained with anti-human ACE2 **(J)**, TMPRSS2 **(K)** or CD147 **(L)** primary antibodies, followed by Cy3-conjugated secondary antibodies (red). Nuclei were stained in blue with DAPI. Images were acquired with a Leica DM 3000 fluorescence microscope using a Leica DFC 7000 camera. Scale bar, 50 μm.

In order to further assess the expression of SARS-CoV-2 receptors, we measured their mRNA and protein levels in different placental cell lines: HUVECs, the immortalized cytotrophoblast cell line HTR8/SVneo, and two trophoblast-derived choriocarcinoma cell lines, namely JAR and BeWo. We initially assessed *ACE2* mRNA expression by RT-qPCR (**Figure 2A**), which showed a particularly high expression in HUVECs. However, the presence of the transcript did not correlate with protein level as demonstrated by Western Blot (WB) analysis (**Figure 2B** and **Supplementary Figure 3**), which revealed the strongest protein expression of ACE2 in JAR. Flow cytometric analysis (**Figure 2C** and **Supplementary Figure 4A**) confirmed WB results, with a higher percentage of ACE2 positive cells in JAR population, both in live and fixed cells (**Supplementary Figure 4D**). On the contrary, the positivity of BeWo and HTR8SVneo emerged only after fixation (**Supplementary Figure 4D**).

The above-mentioned inconsistency between mRNA and protein expression levels was observed for TMPRSS2 as well. **Figure 2D** shows a moderate expression of *TMPRSS2* transcript in JAR and HUVECs, whereas the WB analysis highlights a strong synthesis of the co-receptor by HTR8SVneo (**Figure 2E** and **Supplementary Figure 3**). In this case, the FACS analysis also showed different results as compared to WB, indicating JAR as the major producer of TMPRSS2 (**Figure 2F** and **Supplementary Figure 4B**). Briefly, all the cell lines had low levels of TMPRSS2 protein expression (**Figure 2F**).

The analysis of CD147 mRNA and protein expression (**Figure 2G–I**) confirmed previous observations in IHC. The protein was highly expressed by all the cell lines, especially by JAR and BeWo (**Figures 2H, I** and **Supplementary Figure 4C**). The expression of ACE2, TMPRSS2 and CD147 proteins was also analyzed by immunofluorescence (IF) (**Figures 2J–L**). Consistent with IHC, a positive staining for ACE2 in syncytiotrophoblast cells (BeWo) was noted; accordingly, BeWo and JAR cells showed a low positivity for TMPRSS2 and a high expression of CD147.

### The expression pattern of ACE2 in placenta is modulated by SARS-CoV-2

To gauge the likely mechanism of SARS-CoV-2 infection of placenta, we initially investigated the presence and distribution of the SARS-CoV-2 Spike protein (S2) in placental tissues derived from eight SARS-CoV-2+ pregnant women. Spike protein was identified with variable intensity of staining among the tissue samples (**Supplementary Figures 1B**, **5**).

**Figure 3:**
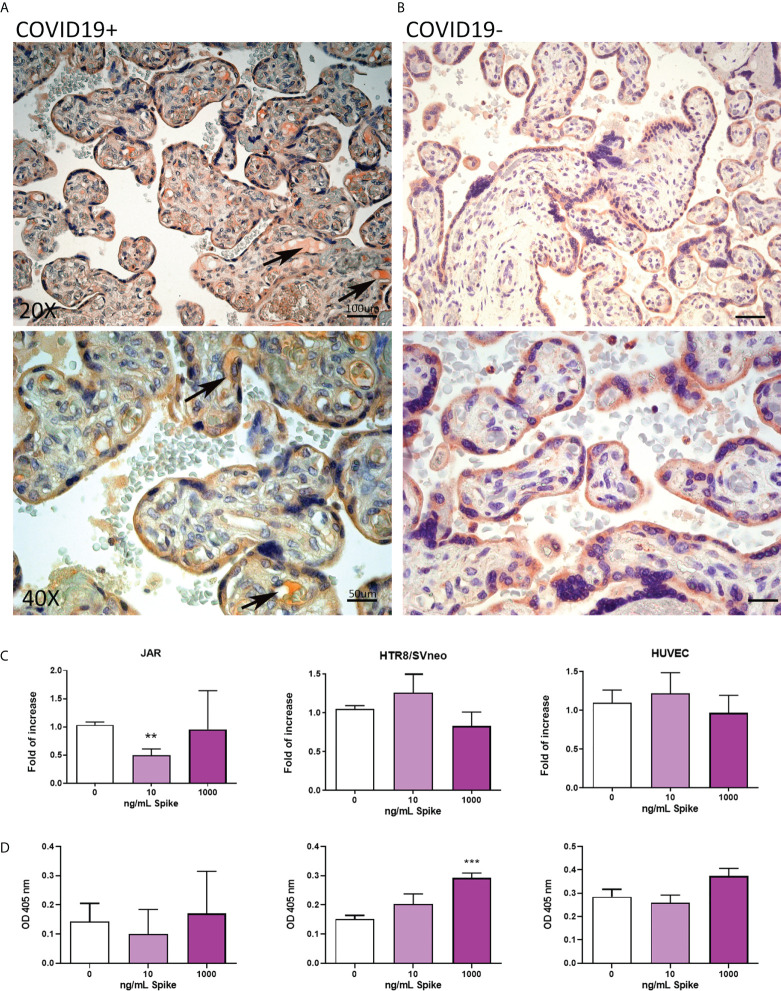
**(A, B)** Representative microphotographs showing expression of ACE2 in COVID-19+ **(A)** or COVID-19- **(B)** placental tissues. Arrows show the presence of ACE2 positivity in fetal vessels of chorionic villi. Streptavidin–biotin–peroxidase complex system with AEC (red) chromogen. **(C)** After stimulation with 10 ng/mL or 1000 ng/mL of Spike S1 protein for 24 h, RNA was extracted from placental cell lines and quantified for *ACE2* expression by RT-qPCR. The expression was normalized using the housekeeping genes *18S, ACTB* and *GAPDH*; results were mediated (geometric mean) and expressed as *fold of increase*. Data are expressed as mean ± SE of three independent experiments performed in triplicate. **p < 0.01, as compared to untreated cells (Mann-Whitney test). **(D)** ELISA on whole cells for detecting ACE2 cell surface expression after 24 h of incubation with 10 ng/mL or 1000 ng/mL of Spike S1 protein. Data are expressed as mean ± SD of two independent experiments performed in triplicate. ***p < 0.001, as compared to untreated cells (Mann-Whitney test).

To characterize the expression of SARS-CoV-2 receptors in the placenta due to COVID-19, we analyzed the pattern of ACE2, TMPRSS2 and CD147 in placental tissues derived from eight SARS-CoV-2+ pregnant patients, and comparing them with seven healthy placentae (**Supplementary Figure 1**). ACE2 staining was localized to the cytoplasm of syncytiotrophoblast cells in normal tissues, whereas SARS-CoV-2+ placentae additionally showed ACE2 positivity in fetal vessels of chorionic villi, as shown in **Figures 3A, B**. As a general trend, we noticed an inverse correlation between the intensity of Spike protein staining and ACE2, even though a statistically significant quantification of this observation was hindered by the low number of samples available (**Supplementary Figures 1B**, **6**).

To mimic *in vitro* the pathologic condition, we stimulated the placental cell lines with Spike protein (S1) at the concentration of 10 or 1000 ng/ml for 24h. After stimulation, ACE2 mRNA and protein expressions in placental cell lines were analyzed by RT-qPCR (**Figure 3C**) and live-cell ELISA (**Figure 3D**). We observed a significant downregulation in *ACE2* mRNA levels only in JAR cells treated with 10ng/ml of Spike protein, whereas the protein expression was upregulated in HTR8/SVneo, although the signal was very low.

Surprisingly, we failed to detect the presence of membrane-bound ACE2 on BeWo cells by ELISA, probably due to the intracellular localization of the epitope recognized by the antibody; in this cell line, we found a significant downregulation of *ACE2* mRNA expression induced by Spike protein at the concentration of 1000 ng/ml (**Supplementary Figure 7**).

### The expression of TMPRSS2 is upregulated in placenta and in placental cells by SARS-CoV-2

**Figure 4:**
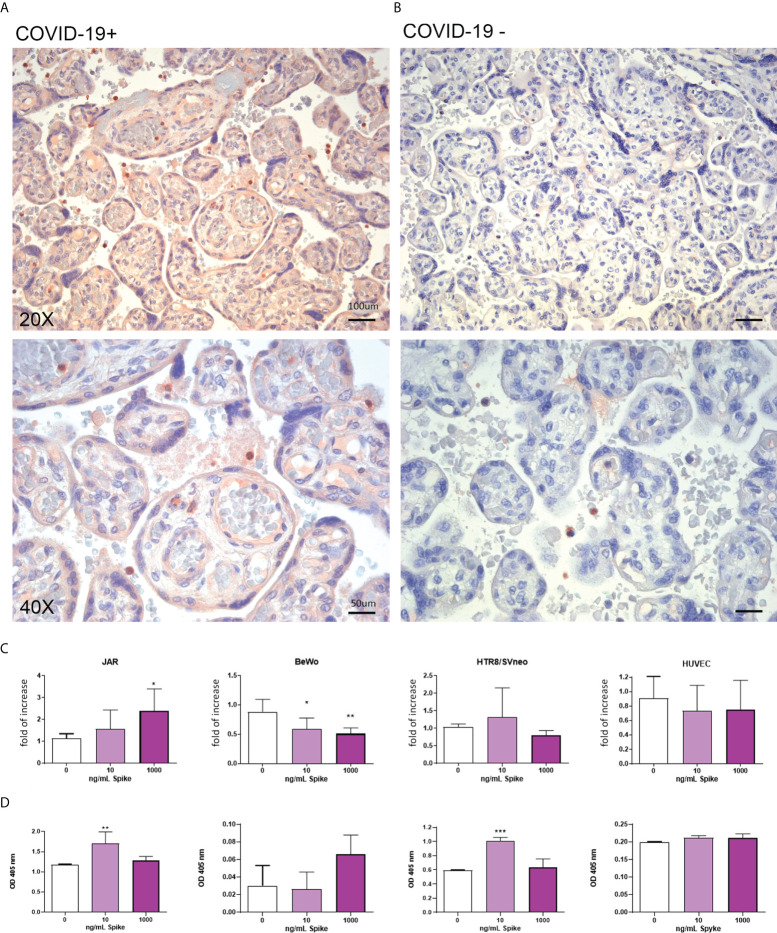
**(A, B)** Representative microphotographs showing the expression of TMPRSS2 in COVID-19+ **(A)** or COVID-19- **(B)** placental tissues. Streptavidin–biotin–peroxidase complex system with AEC (red) chromogen. **(C)** After stimulation with 10 ng/mL or 1000 ng/mL of Spike S1 protein for 24 h, RNA was extracted from placental cell lines and quantified for *TMPRSS2* expression by RT-qPCR. The expression was normalized against the housekeeping genes *18S, ACTB* and *GAPDH*; results were mediated (geometric mean) and expressed as *fold increase*. Data are expressed as mean ± SE of three independent experiments performed in triplicate. *p < 0.05; **p < 0.01, ***p < 0.001, as compared to untreated cells (Mann-Whitney test). **(D)** ELISA on whole cells for detecting TMPRSS2 surface expression following 24 h of incubation with 10 ng/mL or 1000 ng/mL of Spike S1 protein. Data are expressed as mean ± SD of two independent experiments performed in triplicate. *p < 0.05, ***p < 0.001 with respect to untreated cells (Mann-Whitney test).

The level of TMPRSS2 in infected and uninfected placental tissues was evaluated by IHC. As shown in **Figures 4A, B** and **Supplementary Figure 1**, the TMPRSS2 level in SARS-CoV-2+ placental tissues was upregulated compared to placentae from uninfected mothers. Its localization was not limited to the syncytio- and cytotrophoblast, but also in the endothelium of villi vessels. *In vitro* culture of placental cell lines stimulated with the Spike protein validated these observations: trophoblast cells (JAR and HTR8/SVneo) showed an upregulation of TMPRSS2 expression following Spike protein challenge (**Figures 4C, D**). Surprisingly, BeWo cells, when challenged with Spike protein, showed a significant downregulation of *TMPRSS2* mRNA expression.

### Expression of CD147 in placenta is upregulated by SARS-CoV-2

**Figure 5:**
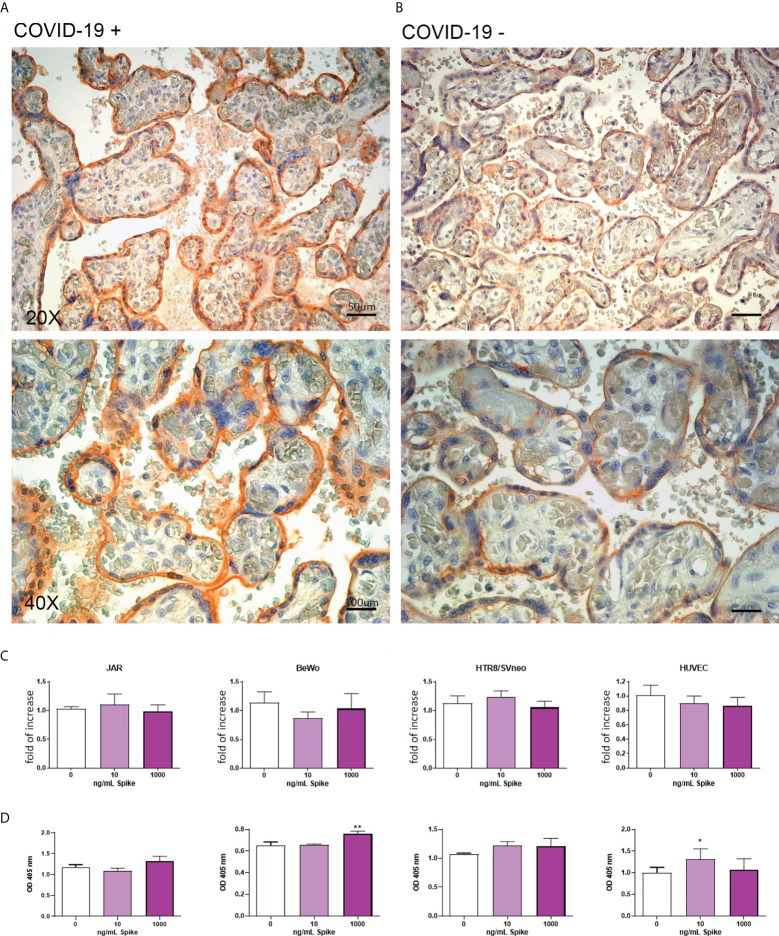
**(A, B)** Representative microphotographs showing the expression of CD147 in COVID-19+ **(A)** or COVID-19- **(B)** placental tissues. Streptavidin–biotin–peroxidase complex system with AEC (red) chromogen. **(C)** After stimulation with 10 ng/mL or 1000 ng/mL of Spike S1 protein for 24 h, RNA was extracted from placental cell lines and quantified for *CD147* expression by RT-qPCR. The expression was normalized using the housekeeping genes *18S, ACTB* and *GAPDH*; results were mediated (geometric mean) and expressed as *fold increase*. Data are expressed as mean ± SE of three independent experiments performed in triplicate. *p < 0.05, **p < 0.01 as compared to untreated cells (Mann-Whitney test). **(D)** ELISA on whole cells for detecting CD147 cell surface expression following 24 h of incubation with 10 ng/mL or 1000 ng/mL of Spike S1 protein. Data are expressed as mean ± SD of two independent experiments performed in triplicate. *p < 0.05, **p < 0.01, as compared to untreated cells (Mann-Whitney test).

The IHC analysis revealed an abundance of CD147 in infected placentae compared to uninfected ones (**Figure 5** and **Supplementary Figure 1**). *In vitro* evaluation of CD147 protein showed an upregulation induced by Spike protein in BeWo and HUVECs cells (**Figure 5**).

### Evaluation of pathogenetic mechanisms of Spike protein

**Figure 6:**
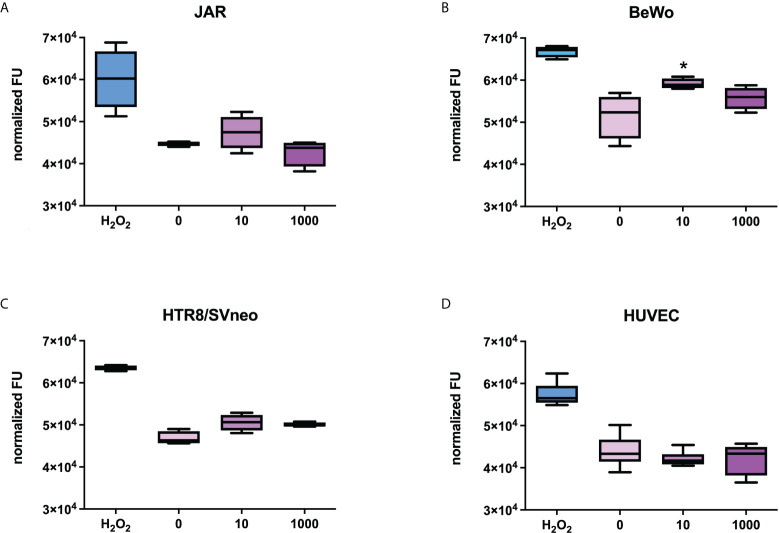
Evaluation of the proapoptotic effect of Spike S1 protein on placental cells. JAR **(A)**, BeWo **(B)**, HTR8/SVneo **(C)** or HUVECs **(D)** were grown to 80% confluence in 96-well plates and incubated with 10 ng/mL or 1000 ng/mL of Spike S1 protein. H_2_O_2_ (0.5 µM) was used as a positive control. The cells were incubated with 5 µM of CellEvent Caspase-3/7 Green Detection Reagent. Fluorescence signals were normalized for the total proteins present in each well. For each group, the line in the middle of the box represents the median. The lower and the upper edges of the box indicate the 1^st^ and 3^rd^ quartile, respectively. Data are results from three independent experiments performed in triplicate. ^∗^p < 0.05, as compared to untreated cells (Mann-Whitney test).

Since the PE-like syndrome is characterized by an increase in syncytiotrophoblast apoptosis, we stimulated the placental cell lines with Spike protein (S1) at the concentration of 10 or 1000 ng/ml for 24h and explored the potential pro-apoptotic effect of the Spike protein on the cells, using a specific assay that detects the activation of caspase 3/7. Hydrogen peroxidase was used as a positive control for apoptosis. As shown in **Figure 6**, BeWo cells showed a slight apoptosis induction with 10 ng/ml of S1 protein, whereas HTR8SVneo required a higher concentration of Spike protein to induce apoptosis. No effects were detected in the other cell populations.

### Analysis of the capability of SARS-CoV-2 to infect placental cells

**Figure 7:**
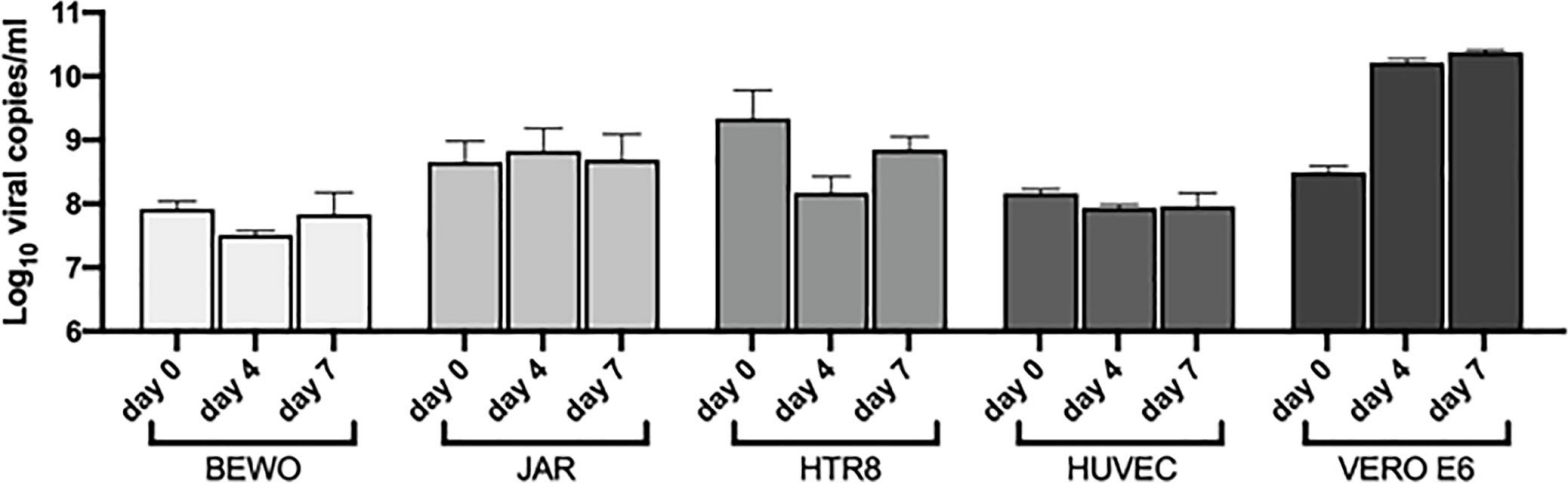
Quantification by RT-qPCR of SARS-CoV-2 RNA presence in the supernatant of cells incubated with the virus. VERO E6 were used as a control. The viral load was displayed as Log_10_ viral copies/ml (mean ± SD) at day 0, 4 and 7 post infection.

BeWo, JAR, HTR8/Svneo and 5 different populations of HUVECs were challenged with SARS-CoV-2 to assess their susceptibility to viral entry and replication. No morphological cytopathic as well as cytotoxic effects (analyzed by MTT assays) were evident in the cell lines during the 7 days of infection. The RNA viral load was monitored during the infection period, but it remained at the basal level recorded on the day 0 (**Figure 7**). Vero E6 cell line was used as a positive control of successful infection, observing an increment in the viral RNA levels as compared to placental cell lines (**Figure 7**). Since a residual quantity of viral RNA was detected in the supernatants, after washing and medium changing, the presence of SARS-CoV-2 was assessed intracellularly using immunofluorescence for the viral nucleocapsid protein; no signal was detected inside the infected cells as compared to Vero E6 cells, where a diffuse staining was detected (**Supplementary Figure 8**).

The cells were also challenged with SARS-CoV-2 for 24 hours and then incubated with new virus to determine if the pre-treatment could enhance the susceptibility of the cells to the infection. Despite the pre-stimulation, no viral replication was detected after 4 days of infection (**Supplementary Figure 9**), suggesting that placental cells were resistant to SARS-CoV-2 entry *in vitro*.

### Evaluation of the mechanisms of resistance to infection

**Figure 8:**
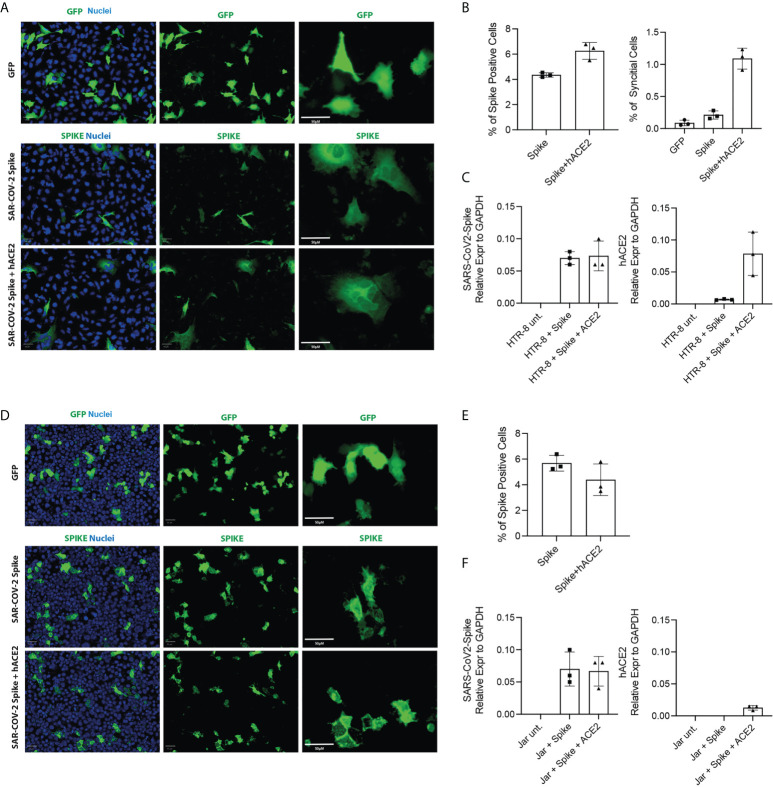
Cell Fusion Assay. HTR8/SVneo and JAR cells were seeded 10h before transfection. Cells were then transfected with pCMV-SPIKEDelta-V5+pCMV-hACE2, pCMV-SPIKEDelta-V5+pcDNA3 or pCMV-GFP+pcDNA3. Representative images of green fluorescence protein (GFP) and immunostaining for SARS-CoV-2 -Spike in HTR8 **(A)** and JAR **(D)** taken using the Operetta high content screening microscope (PerkinElmer) with Olympus 20 x (NA-0.45) objective. Quantification of the total cells expressing Spike and the total number of syncytia per well in HTR8 **(B)** and JAR **(E)**. mRNA expression levels by RT-qPCR of SARS-CoV2-Spike and hACE2 in HTR8 **(C)** and JAR **(F)**.

In order to understand the mechanisms underlying the resistance to SARS-CoV-2 infection by placental cells, we performed transfection with the Spike protein to observe the formation of cellular syncytia. The simultaneous presence of Spike protein and functional receptor-co-receptor combination (ACE2, TMPRSS2) on the cell surface, in fact, led to the activation of fusogenic mechanisms which manifested in the formation of syncytia (**Figure 8**). Since BeWo cells, by their nature, tend to form syncytia, they were excluded from the analysis. Our results indicated that HTR8/SVneo were more amenable to form cellular syncitia even under basal conditions (**Figures 8A, B**), whilst JAR cells overexpressing the human ACE2 gene together with SARS-CoV-2 Spike were unable to form syncytial structures at 24 hours post-transfection (**Figure 8D**), despite showing a similar number of cells positive for SARS-CoV-2-Spike (**Figure 8E**) as compared to HTR8/SVneo cells. HTR8/SVneo exhibited increase in syncytial structures upon SARS-CoV-2-Spike transfection alone and together with humanACE2 (**Figure 8B**). Expression levels of SARS-CoV-2-Spike and human ACE2 were assessed by RT-qPCR at 24 hours post-transfection (**Figures 8C, F**). Vero E6 cell line was used as a positive control (**Supplementary Figure 10**).

### SARS-CoV-2 induced pro-inflammatory cytokine expression in syncytiotrophoblast cells

**Figure 9:**
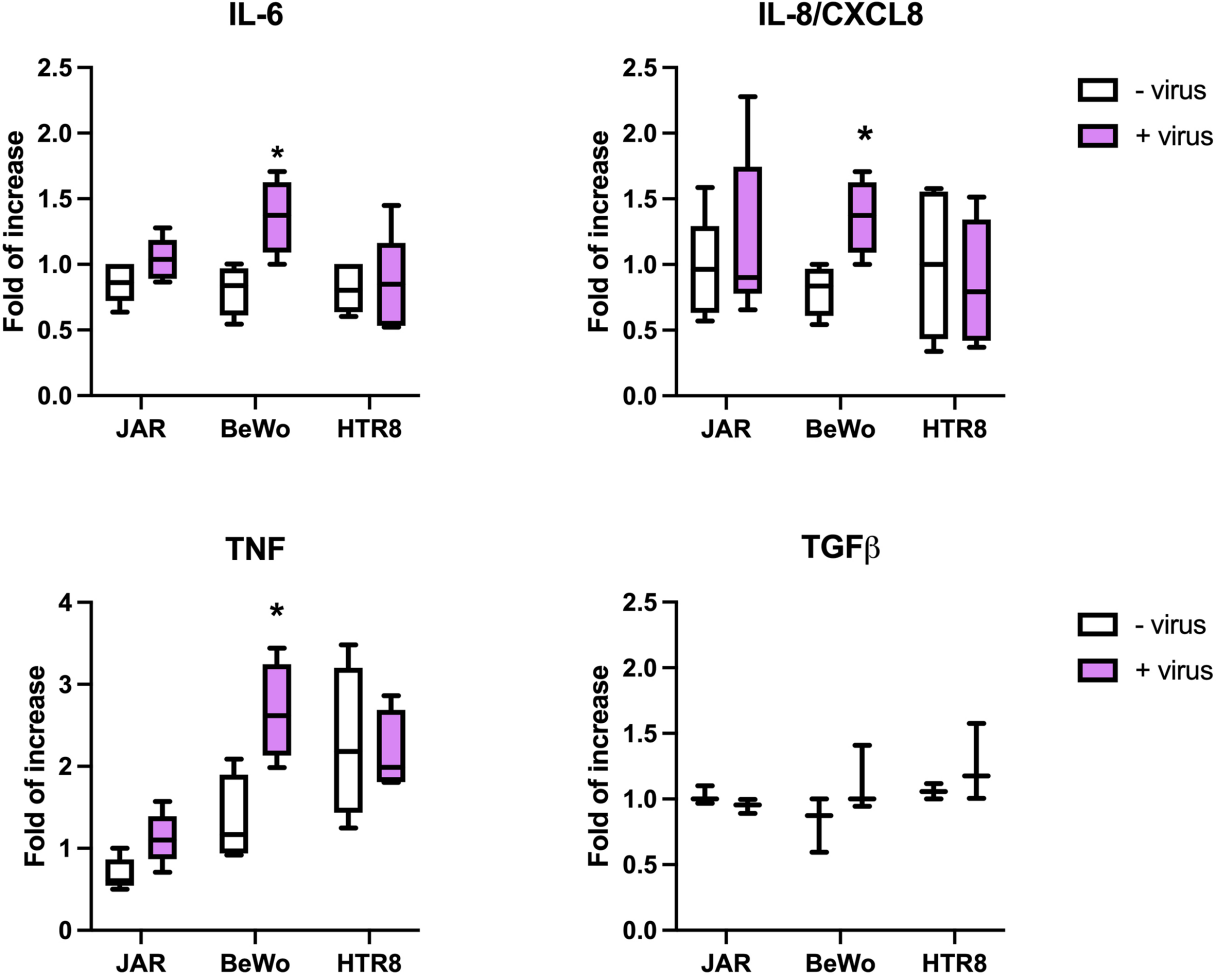
Evaluation of cytokine expression induced by SARS-CoV-2. The placental cells were challenged for 24 hours with SARS-CoV-2; total RNA was isolated and analyzed by RT-qPCR for the expression of IL-6, IL-8, TNF-α and TGF-β. The expression was normalized against the housekeeping genes *18S, ACTB* and *GAPDH*; results were mediated and expressed as *fold increase*. Data are expressed as mean ± SD of two independent experiments performed in triplicate. *p < 0.05.

We next investigated the direct activation induced by intact SARS-CoV-2 in terms of cytokine expression. The placental cells were incubated for 24h with the virus and the cytokine gene expression was investigated by RT-qPCR. As shown in **Figure 9**, the virus-cell contact induced the upregulation of IL-6, IL-8 and TNF-α in BeWo cells. These data were confirmed by the incubation of placental cells with Spike protein only, as shown in **Supplementary Figure 11**.

### Evaluation of the effects of SARS-CoV-2 and spike protein on endothelial cell activation

**Figure 10:**
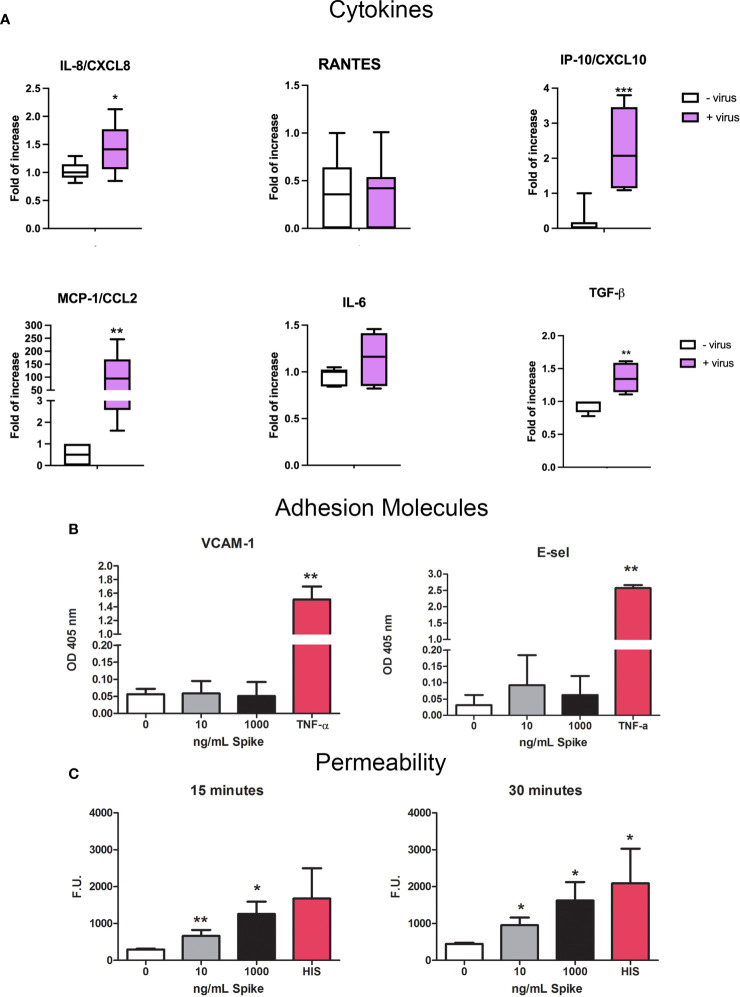
Pro-inflammatory effect of SARS-CoV-2 or Spike S1 protein on HUVECs. **(A)** RT-qPCR for gene expression analysis of MCP-1/CCL2, RANTES/CCL5, IP-10/CXCL10, IL-8/CXCL8, IL-6 and TGF-β in SARS-CoV-2-stimulated HUVECs. After 24 h of treatment with S1, total mRNA was isolated and gene expression analysis was performed by RT-qPCR. The expression was normalized using the housekeeping genes *18S, ACTB and GAPDH*; the results were mediated and expressed as *fold increase*. Data are expressed as mean ± SD of two independent experiments performed in triplicate. *p < 0.05; **p < 0.01; ***p < 0.001, as compared to untreated cells. **(B)** Analysis of the expression of the adhesion molecules VCAM-1 and E-Selectin. Four different populations of HUVECs were grown to confluence in a 96-well plate and incubated with 10 ng/mL or 1000 ng/mL of Spike S1 protein. TNF-α (100 ng/mL) was used as a positive control. Cells were incubated with anti-human VCAM-1 or anti-human E-selectin monoclonal antibodies, followed by alkaline phosphatase-conjugated secondary antibodies. Data are expressed as mean ± SE of four experiments performed in triplicate. **p < 0.01, as compared to untreated cells (Mann-Whitney test). **(C)** Permeabilizing effect of Spike protein on endothelial cells. The permeabilizing activity was evaluated kinetically, after 15 and 30 minutes, by adding 10 ng/mL or 1000 ng/mL of Spike protein to the upper chamber of the transwell, and then measuring the amount of FITC-labeled BSA that leaked through a monolayer of endothelial cells into the lower chamber. Histamine (HIS) was used as a positive control. Data are expressed as mean ± SD of four experiments performed in duplicate. *p < 0.05; **p < 0.01, as compared to untreated cells (Mann-Whitney test).

Since SARS-CoV-2 infection is responsible for endothelial dysfunction and disruption of vascular integrity, resulting in hyperinflammation and hypercoagulability state (39, 40), we investigated the potential endothelial damage/activation effect on HUVECs. First, we analyzed if the virus could induce the expression of pro-inflammatory/pro-fibrotic chemokines/cytokines. Our results confirmed the role of ECs in cytokine storm; SARS-CoV-2-challenged HUVECs induced a strong upregulation of MCP-1/CCL2 expression and, to a lesser extent, IP-10/CXCL10, IL-8 and TGF-β (**Figure 10A**).

We also investigated the expression of adhesion molecules VCAM-1 and E-Selectin by HUVECs. We stimulated the cells with 10 or 1000 ng/mL of S1 protein for 18h or 4h respectively and then performed ELISA on whole live cells. TNF-α (100 ng/mL) stimulation was used as a positive control. No exposure of VCAM-1 nor E-Selectin was reveled after Spike stimulation by HUVECs, compared to TNF-α-stimulation (**Figure 10B**). Moreover, we performed permeability assay in order to evaluate endothelial monolayer integrity. The results of leakage assay, as shown in **Figure 10C**, indicated that Spike protein (10 and 1000 ng/mL) was able to increase the permeability with a statistically significant effect after 15 and 30 minutes.

## Discussion

SARS-CoV-2 is a novel and deadly coronavirus causing global health concerns among pregnant women. Two years after the outbreak of the pandemic, the maternal-neonatal vertical transmission mechanisms of COVID-19 are yet to be clarified. The association between SARS-CoV-2 infection in pregnancy and the onset of gestational complications are quite well-established (8, 9, 15). Despite isolated cohorts showing a high percentage of vertical transmission, no universal consensus has been reached yet. More recent meta-analyses showed that about 2-3% of newborns from SARS-CoV-2 positive women were infected (3, 4). Since SARS-CoV-2 positivity in the new-born was shown to be associated with maternal factors (severe COVID 19 manifestations, death and admission to intensive care unit) (4), the variability among different studies may be mainly due to cohort selection and inclusion criteria. In any case, the mechanisms for vertical transmission and the onset of gestational complications are not fully understood.

In the current study, we assessed the expression of SARS-CoV-2 entry receptors in the normal placenta and investigated how this expression was modulated by the virus. It is well established that the infection of host cells by SARS-CoV-2 requires the presence of two receptors: ACE2 and TMPRSS2 (16). However, SARS-CoV-2 may bind to a third alternative receptor, the CD147/EMMPRIN/BSG (18), a membrane protein belonging to the immunoglobulin superfamily that has been implicated in tissue remodeling and in pathological conditions such as atherosclerosis, aneurysm, heart failure, osteoarthritis, and cancer (20, 41–43). Among the different receptors identified for the entry of SARS-CoV-2, CD147 is an inducer of matrix metalloproteinases and angiogenic factors, and is expressed in the uterus having an important role in embryo implantation (44). It is also one of the key surface molecules involved in regulating invasion and differentiation of human trophoblast (19).

To elucidate the mechanisms of SARS-CoV-2 infection of the placenta, we first evaluated the basal expression and distribution of entry receptors by performing IHC on healthy placentae. ACE2 expression was localized on the syncytiotrophoblast monolayer, although we noticed highly variable intensity among patients, consistent with earlier reports (29, 45, 46). The staining for TMPRSS2 was either very weak or absent, with the exception of some isolated cells in the intervillous space, which are likely to be polymorphonuclear leukocytes, confirming the observations reported in recent studies (26, 27, 47). On the contrary, the intensity of CD147 staining was strong mainly in the brush border of syncytiotrophoblasts, as reported by others (19, 48, 49). We also evaluated the local gene expression of the receptors, by performing RT-qPCR on healthy placental tissues and on isolated primary placental cells. Several studies have analyzed SARS-CoV-2 entry receptors in placenta by molecular pathology methods and placenta-derived cells from a publicly available database and analyzed by bioinformatics (26, 27), reporting that ACE2 and TMPRSS2 are expressed in the placenta during the first trimester, which diminishes across gestational age (28). These data are partly in contrast to our observation regarding the mRNA expression of the receptors. Moreover, previous studies indicated that there was no co-expression or localization of both receptors in the placenta at term (27, 50). Our results showed that EVT, DECs and DSCs expressed a great amount of ACE2 and CD147 transcripts, whereas TMPRSS2 was expressed only by DSCs and, to a lesser extent, by EVT. In 2005, Valdés *rom* (29) showed that, in placental villi, the main sites of ACE2 expression were the syncytiotrophoblast, cytotrophoblast and endothelium; in decidua, ACE2 was expressed in the invading and intravascular trophoblast as well as in arterial and venous endothelium of the umbilical cord. Tagluaer and coworkers identified the abundance of ACE2 expression in comparison with TMPRSS2 (26); virus protein and ACE2 expression consistently localized primarily within the outer syncytiotrophoblast layer of placental villi.

To perform *in vitro* functional assays, we used the placental cell lines JAR, HTR8/SVneo and BeWo, as models of cytotrophoblast and syncytyotrophoblast cells, and HUVECs, the endothelial cells isolated from human umbilical cord vein, as a model of villi endothelium. We performed RT-qPCR, flow cytometry, IF and WB assays, to validate the IHC data: the expression of TMPRSS2 was very low, whereas that of CD147 appeared to be greater. In general, JAR, BeWo and HUVECs showed ACE2 expression.

We performed IHC analyses of the entry receptors on SARS-CoV-2 infected placentae and compared them with healthy placentae. Moreover, to mimic *in vitro* the effect of SARS-CoV-2 presence in the placenta, we stimulated the cells with Spike protein (S1). A significant pro-apoptotic effect was observed, in BeWo and HTR8/SVneo cell lines, but not in HUVECs and JAR cells. Mourad *et al* found that the expression level of placental ACE2, but not TMPRSS2, was higher in women with severe COVID-19 (51). However, the study did not analyze infected placentae; only placenta presenting SARS-CoV-2 RNA expressed high levels of TMPRSS2. In our study, stimulation of placental cells with Spike protein resulted in an upregulation of TMPRSS2 in cytotrophoblast cell lines (JAR and HTR8/SVneo), whereas no effects were observed on HUVEC. These results were partially in accordance with IHC analysis of SARS-CoV-2 positive placentae that revealed an increase in TMPRSS2 staining. A significant downregulation of ACE2 mRNA expression was observed in JAR and BeWo; however, this was not confirmed by protein analysis. We also noticed an increase in CD147 staining of SARS-CoV-2 infected placentae compared to non-infected counterparts. Spike protein was able to enhance the expression of CD147 at protein level in all placental cell lines (JAR and BeWo) and HUVEC.

In view of the conflicting reports on the ability of SARS-CoV-2 to infect placental cells SARS-CoV-2 to infect placental cells, since contradictory (52, 53), we challenged the placental cell lines with SARS-CoV-2. No viral replication was observed suggesting that BeWo, JAR, HTR8/SVneo and HUVECs were not permissive to viral infection. SARS-CoV-2 was also not able to enter the cells, since the immuno staining for the viral nucleocapsid protein showed no positivity, and no morphological alterations were observed. Since the treatment of the cells with Spike protein enhanced the expression of ACE2, TMPRSS2 and CD147, cells were first primed with the virus, then new virus was added. However, no viral replication was observed after 4 days. EC infection with SARS-CoV-2 has been reported under well-defined conditions of culture, such as 3D culture and *in vivo* studies (54, 55). It is possible that the dysfunction of EC glycocalyx induced by ROS production by SARS-CoV-2-activated leukocytes could facilitate the entry of the virus into ECs.

We carried out transfection with the Spike protein to view the formation of cellular syncytia because the simultaneous presence of Spike and functional co-receptors on the cell surface leads to the activation of fusogenic mechanisms which manifest themselves in the formation of syncytia (56). We found that JAR cells, co-transfected with human ACE2 and SARS-CoV2-Spike constructs, did not form syncytial structures at 24 hours after transfection. As suggested earlier (19, 57) IFN-induced transmembrane proteins (IFITMs), a family of restriction factors blocking the entry step of many viruses, could be involved in the resistance to syncytia formation by trophoblast cells.

Since COVID-19 is characterized by cytokine storm (25, 26), we investigated induction of pro- and anti-inflammatory cytokines by placental cells when challenged with intact SARS-CoV-2 or Spike protein. In particular, BeWo cells showed an upregulation of IL-6, IL-8 and TNF-α when challenged with the intact virus, but they appeared to be less responsive to Spike protein, probably due to the proapoptotic effect induced by the virus protein on these cells. S1 Spike protein on its own was also sufficient to cause increased pro-inflammatory cytokine production: in particular, IL-6 expression was found to be significantly higher in JAR cells challenged with the higher concentration of Spike protein. Thus, placental cells contribute to the cytokine storm induced by SARS-CoV-2 (58). Interestingly, IL-8, a pro-angiogenic factor, was significantly upregulated in HUVECs challenged with the higher concentration of Spike protein.

Evidence that COVID-19 is not merely a pulmonary disease is currently accumulating. In fact, SARS-CoV-2 has a great tropism for several different organs due to wide distribution of ACE2, making COVID-19 a multi-organ disease. One of its main clinical manifestations is endothelial cell dysfunction, causing systemic vasculitis and thrombotic complications (59–61). Among the multiple mechanisms leading to endothelial cell dysfunction, an increase in adhesion molecule expression and vascular permeability in the pulmonary ECs have been reported (40). We therefore investigated the ability of HUVECs, a classical endothelial model system, to respond to Spike stimulation. We first confirmed the pivotal role of ECs in cytokine storm: in fact, SARS-CoV-2 induced a strong upregulation of MCP-1/CCL2 expression, and to a lesser extent, of IP-10/CXCL10, IL-8 and TGF-β in HUVECs. Noteworthy is the up-regulation of TGF-β synthesis, a cytokine considered anti-inflammatory but notoriously involved in fibrosis, a pivotal pathogenetic process in COVID-19. Since SARS-CoV-2 infection is responsible for endothelial dysfunction, we analyzed the ability of the virus to induce activation of EC. However, S1 protein did not alter the adhesion molecules VCAM-1 and E-Selectin exposure by HUVECs. Nevertheless, S1 protein was able to alter endothelial monolayer integrity and make it leakier by increasing cell permeability.

A limitation of this study is that the observations are primarily based on the use of trophoblast cell lines and not on primary trophoblasts isolated from human placentas. Although the cell lines are very useful for obtaining a simple, rapid and reproducible overview of the behavior of the placenta, the results obtained with these lines have not always been confirmed on the primary cells. Another limiting component is that not all the known receptors for the entry of SARS-CoV-2, which would have given a better overview of the possibility of the virus infecting the placental cells, have been examined.

In conclusion, our study revealed that healthy non-infected placentae have elevated levels of CD147 expression and variable levels of ACE2, while TMPRSS2 is present in a very low quantity. Thus, healthy placenta is a non-preferential tissue for SARS-CoV-2 infection. COVID-19 and the presence of virus can up-regulate TMPRSS2, facilitating the virus entry into syncytiotrophoblast and cytotrophoblast cells. We also demonstrate that trophoblast cell lines and endothelial cells are not permissive to virus entry, probably due to a resistance to the fusogenic process or glycocalyx presence respectively. The systemic effect linked to endotheliitis, and therefore development of the preeclampsia-like syndrome in the infected mothers could be due to the direct effect of the spike protein (with its shedding and release) on placental cells, which in turn, can cause increased expression of pro-inflammatory cytokines, increased vascular permeability, and induction of syncytiotrophoblast apoptosis.

## Data availability statement

The original contributions presented in the study are included in the article/**Supplementary Material**. Further inquiries can be directed to the corresponding author.

## Ethics statement

The studies involving human participants were reviewed and approved by Institutional Review Board of The Maternal-Children’s Hospital Burlo Garofolo. The patients/participants provided their written informed consent to participate in this study.

## Author contributions

Conceptualization: CA and RB. Methodology: MT, MS, AB, LZ, and SM. Resources: GZ, AM, TS, FR and FF. Data curation: CA, MT and AB. Writing—original draft preparation: CA, MT and MS. Writing—review and editing: CA, AB, AM, LB, UK and RB. Supervision: LB, SC, MC, GR and RB. Funding acquisition: CA, GR and RB. All authors have read and agreed to the published version of the manuscript. All authors contributed to the article and approved the submitted version.

## Funding

This research was supported by Ferring COVID-19 Investigational Grant (GRAVISAR to RB). The funder Ferring Pharmaceuticals was not involved in the study design, collection, analysis, interpretation of data, the writing of this article or the decision to submit it for publication. This work was supported by the Ministry of Health, Rome - Italy, in collaboration with the Institute for Maternal and Child Health IRCCS Burlo Garofolo, Trieste – Italy (RC24/19 and 47/20 to GR and 40/20 and 09/21 to CA).

## Conflict of interest

The authors declare that the research was conducted in the absence of any commercial or financial relationships that could be construed as a potential conflict of interest.

## Publisher’s note

All claims expressed in this article are solely those of the authors and do not necessarily represent those of their affiliated organizations, or those of the publisher, the editors and the reviewers. Any product that may be evaluated in this article, or claim that may be made by its manufacturer, is not guaranteed or endorsed by the publisher.

## References

[1] Varghese PM Tsolaki AG Yasmin H Shastri A Ferluga J Vatish M. Host-pathogen interaction in COVID-19: Pathogenesis, potential therapeutics and vaccination strategies. Immunobiology (2020) 225(6):152008. doi: 10.1016/j.imbio.2020.152008 33130519 PMC7434692

[2] Yasmin H Saha S Butt MT Modi RK George AJT Kishore U. SARS-CoV-2: Pathogenic mechanisms and host immune response. Adv Exp Med Biol (2021) 1313:99–134. doi: 10.1007/978-3-030-67452-6_6 34661893

[3] Kotlyar AM Grechukhina O Chen A Popkhadze S Grimshaw A Tal O. Vertical transmission of coronavirus disease 2019: A systematic review and meta-analysis. Am J Obstet Gynecol (2021) 224(1):35–53 e3. doi: 10.1016/j.ajog.2020.07.049 32739398 PMC7392880

[4] Allotey J Chatterjee S Kew T Gaetano A Stallings E Fernandez-Garcia S. SARS-CoV-2 positivity in offspring and timing of mother-to-child transmission: living systematic review and meta-analysis. BMJ (2022) 376:e067696. doi: 10.1136/bmj-2021-067696 35296519 PMC8924705

[5] Di Mascio D Khalil A Saccone G Rizzo G Buca D Liberati M. Outcome of coronavirus spectrum infections (SARS, MERS, COVID-19) during pregnancy: a systematic review and meta-analysis. Am J Obstet Gynecol MFM (2020) 2(2):100107. doi: 10.1016/j.ajogmf.2020.100107 32292902 PMC7104131

[6] Zaigham M Andersson O. Maternal and perinatal outcomes with COVID-19: A systematic review of 108 pregnancies. Acta Obstet Gynecol Scand (2020) 99(7):823–9. doi: 10.1111/aogs.13867 PMC7262097 32259279

[7] Azinheira Nobrega Cruz N Stoll D Casarini DE Bertagnolli M. Role of ACE2 in pregnancy and potential implications for COVID-19 susceptibility. Clin Sci (Lond) (2021) 135(15):1805–24. doi: 10.1042/CS20210284 PMC8329853 34338772

[8] Di Toro F Gjoka M Di Lorenzo G De Santo D De Seta F Maso G. Impact of COVID-19 on maternal and neonatal outcomes: A systematic review and meta-analysis. Clin Microbiol Infect (2021) 27(1):36–46. doi: 10.1016/j.cmi.2020.10.007 33148440 PMC7605748

[9] Metz TD Clifton RG Hughes BL Sandoval GJ Grobman WA Saade GR. Association of SARS-CoV-2 infection with serious maternal morbidity and mortality from obstetric complications. JAMA (2022) 327(8):748–59. doi: 10.1001/jama.2022.1190 PMC8822445 35129581

[10] Chen H Guo J Wang C Luo F Yu X Zhang W. Clinical characteristics and intrauterine vertical transmission potential of COVID-19 infection in nine pregnant women: A retrospective review of medical records. Lancet (2020) 395(10226):809–15. doi: 10.1016/S0140-6736(20)30360-3 PMC7159281 32151335

[11] Narang K Enninga EAL Gunaratne M Ibirogba ER Trad ATA Elrefaei A. SARS-CoV-2 infection and COVID-19 during pregnancy: A multidisciplinary review. Mayo Clin Proc (2020) 95(8):1750–65. doi: 10.1016/j.mayocp.2020.05.011 PMC7260486 32753148

[12] Papageorghiou AT Deruelle P Gunier RB Rauch S Garcia-May PK Mhatre M. Preeclampsia and COVID-19: Results from the INTERCOVID prospective longitudinal study. Am J Obstet Gynecol (2021) 225(3):289 e1– e17. doi: 10.1016/j.ajog.2021.05.014 PMC8233533 34187688

[13] Medeiros LT Peracoli JC Bannwart-Castro CF Romao M Weel IC Golim MA. Monocytes from pregnant women with pre-eclampsia are polarized to a M1 phenotype. Am J Reprod Immunol (2014) 72(1):5–13. doi: 10.1111/aji.12222 24689463

[14] Costela-Ruiz VJ Illescas-Montes R Puerta-Puerta JM Ruiz C Melguizo-Rodriguez L. SARS-CoV-2 infection: The role of cytokines in COVID-19 disease. Cytokine Growth Factor Rev (2020) 54:62–75. doi: 10.1016/j.cytogfr.2020.06.001 32513566 PMC7265853

[15] Agostinis C Mangogna A Balduit A Aghamajidi A Ricci G Kishore U. COVID-19, pre-eclampsia, and complement system. Front Immunol (2021) 12:775168. doi: 10.3389/fimmu.2021.775168 34868042 PMC8635918

[16] Hoffmann M Kleine-Weber H Schroeder S Kruger N Herrler T Erichsen S. SARS-CoV-2 cell entry depends on ACE2 and TMPRSS2 and is blocked by a clinically proven protease inhibitor. Cell (2020) 181(2):271–80 e8. doi: 10.1016/j.cell.2020.02.052 32142651 PMC7102627

[17] Bourgonje AR Abdulle AE Timens W Hillebrands JL Navis GJ Gordijn SJ. Angiotensin-converting enzyme 2 (ACE2), SARS-CoV-2 and the pathophysiology of coronavirus disease 2019 (COVID-19). J Pathol (2020) 251(3):228–48. doi: 10.1002/path.5471 PMC7276767 32418199

[18] Wang K Chen W Zhang Z Deng Y Lian JQ Du P. CD147-spike protein is a novel route for SARS-CoV-2 infection to host cells. Signal Transduct Target Ther (2020) 5(1):283. doi: 10.1038/s41392-020-00426-x 33277466 PMC7714896

[19] Lee CL Lam MP Lam KK Leung CO Pang RT Chu IK. Identification of CD147 (basigin) as a mediator of trophoblast functions. Hum Reprod (2013) 28(11):2920–9. doi: 10.1093/humrep/det355 24014600

[20] Yurchenko V Constant S Bukrinsky M. Dealing with the family: CD147 interactions with cyclophilins. Immunology (2006) 117(3):301–9. doi: 10.1111/j.1365-2567.2005.02316.x PMC1782239 16476049

[21] Pushkarsky T Zybarth G Dubrovsky L Yurchenko V Tang H Guo H. CD147 facilitates HIV-1 infection by interacting with virus-associated cyclophilin a. Proc Natl Acad Sci U.S.A. (2001) 98(11):6360–5. doi: 10.1073/pnas.111583198 PMC33473 11353871

[22] Zhou J Zhu P Jiang JL Zhang Q Wu ZB Yao XY. Involvement of CD147 in overexpression of MMP-2 and MMP-9 and enhancement of invasive potential of PMA-differentiated THP-1. BMC Cell Biol (2005) 6(1):25. doi: 10.1186/1471-2121-6-25 15904490 PMC1156878

[23] Kim JY Kim WJ Kim H Suk K Lee WH. The stimulation of CD147 induces MMP-9 expression through ERK and NF-kappaB in macrophages: Implication for atherosclerosis. Immune Netw (2009) 9(3):90–7. doi: 10.4110/in.2009.9.3.90 PMC2803300 20107538

[24] Fuentes-Prior P. Priming of SARS-CoV-2 s protein by several membrane-bound serine proteinases could explain enhanced viral infectivity and systemic COVID-19 infection. J Biol Chem (2021) 296:100135. doi: 10.1074/jbc.REV120.015980 33268377 PMC7834812

[25] Hecht JL Quade B Deshpande V Mino-Kenudson M Ting DT Desai N. SARS-CoV-2 can infect the placenta and is not associated with specific placental histopathology: a series of 19 placentas from COVID-19-positive mothers. Mod Pathol (2020) 33(11):2092–103. doi: 10.1038/s41379-020-0639-4 PMC7395938 32741970

[26] Taglauer E Benarroch Y Rop K Barnett E Sabharwal V Yarrington C. Consistent localization of SARS-CoV-2 spike glycoprotein and ACE2 over TMPRSS2 predominance in placental villi of 15 COVID-19 positive maternal-fetal dyads. Placenta (2020) 100:69–74. doi: 10.1016/j.placenta.2020.08.015 32862058 PMC7445146

[27] Pique-Regi R Romero R Tarca AL Luca F Xu Y Alazizi A. Does the human placenta express the canonical cell entry mediators for SARS-CoV-2? Elife (2020) 9:e58716. doi: 10.7554/eLife.58716 32662421 PMC7367681

[28] Bloise E Zhang J Nakpu J Hamada H Dunk CE Li S. Expression of severe acute respiratory syndrome coronavirus 2 cell entry genes, angiotensin-converting enzyme 2 and transmembrane protease serine 2, in the placenta across gestation and at the maternal-fetal interface in pregnancies complicated by preterm birth or preeclampsia. Am J Obstet Gynecol (2021) 224(3):298 e1– e8. doi: 10.1016/j.ajog.2020.08.055 PMC7445125 32853537

[29] Valdes G Neves LA Anton L Corthorn J Chacon C Germain AM. Distribution of angiotensin-(1-7) and ACE2 in human placentas of normal and pathological pregnancies. Placenta (2006) 27(2-3):200–7. doi: 10.1016/j.placenta.2005.02.015 16338465

[30] Yamaleyeva LM Pulgar VM Lindsey SH Yamane L Varagic J McGee C. Uterine artery dysfunction in pregnant ACE2 knockout mice is associated with placental hypoxia and reduced umbilical blood flow velocity. Am J Physiol Endocrinol Metab (2015) 309(1):E84–94. doi: 10.1152/ajpendo.00596.2014 PMC4490333 25968580

[31] Lanza K Perez LG Costa LB Cordeiro TM Palmeira VA Ribeiro VT. Covid-19: the renin-angiotensin system imbalance hypothesis. Clin Sci (Lond) (2020) 134(11):1259–64. doi: 10.1042/CS20200492 PMC7276636 32507883

[32] Bharadwaj MS Strawn WB Groban L Yamaleyeva LM Chappell MC Horta C. Angiotensin-converting enzyme 2 deficiency is associated with impaired gestational weight gain and fetal growth restriction. Hypertension (2011) 58(5):852–8. doi: 10.1161/HYPERTENSIONAHA.111.179358 PMC3228834 21968754

[33] Vouga M Favre G Martinez-Perez O Pomar L Acebal LF Abascal-Saiz A. Maternal outcomes and risk factors for COVID-19 severity among pregnant women. Sci Rep (2021) 11(1):13898. doi: 10.1038/s41598-021-92357-y 34230507 PMC8260739

[34] Jaffe EA Nachman RL Becker CG Minick CR. Culture of human endothelial cells derived from umbilical veins. identification by morphologic and immunologic criteria. J Clin Invest (1973) 52(11):2745–56. doi: 10.1172/JCI107470 PMC302542 4355998

[35] Agostinis C Rami D Zacchi P Bossi F Stampalija T Mangogna A. Pre-eclampsia affects procalcitonin production in placental tissue. Am J Reprod Immunol (2018) 79(4):e12823. doi: 10.1111/aji.12823 29427369

[36] Fedchenko N Reifenrath J. Different approaches for interpretation and reporting of immunohistochemistry analysis results in the bone tissue - a review. Diagn Pathol (2014) 9:221. doi: 10.1186/s13000-014-0221-9 25432701 PMC4260254

[37] McCurdy RD McGrath JJ Mackay-Sim A. Validation of the comparative quantification method of real-time PCR analysis and a cautionary tale of housekeeping gene selection. Gene Ther Mol Biol (2008) 12(1):1215–24.

[38] Braga L Ali H Secco I Chiavacci E Neves G Goldhill D. Drugs that inhibit TMEM16 proteins block SARS-CoV-2 spike-induced syncytia. Nature (2021) 594(7861):88–93. doi: 10.1038/s41586-021-03491-6 33827113 PMC7611055

[39] Bonaventura A Vecchie A Dagna L Martinod K Dixon DL Van Tassell BW. Endothelial dysfunction and immunothrombosis as key pathogenic mechanisms in COVID-19. Nat Rev Immunol (2021) 21(5):319–29. doi: 10.1038/s41577-021-00536-9 PMC8023349 33824483

[40] Teuwen LA Geldhof V Pasut A Carmeliet P. COVID-19: the vasculature unleashed. Nat Rev Immunol (2020) 20(7):389–91. doi: 10.1038/s41577-020-0343-0 PMC7240244 32439870

[41] Xiong L Edwards CK Zhou L 3rd. The biological function and clinical utilization of CD147 in human diseases: a review of the current scientific literature. Int J Mol Sci (2014) 15(10):17411–41. doi: 10.3390/ijms151017411 PMC4227170 25268615

[42] Landras A Reger de Moura C Jouenne F Lebbe C Menashi S Mourah S. CD147 is a promising target of tumor progression and a prognostic biomarker. Cancers (Basel) (2019) 11(11):1803. doi: 10.3390/cancers11111803 PMC6896083 31744072

[43] Guindolet D Gabison EE. Role of CD147 (EMMPRIN/Basigin) in tissue remodeling. Anat Rec (Hoboken) (2020) 303(6):1584–9. doi: 10.1002/ar.24089 30768865

[44] Li K Nowak RA. The role of basigin in reproduction. Reproduction (2019) 159(2):R97–R109. doi: 10.1530/REP-19-0268 31600731

[45] Malinowski AK Noureldin A Othman M. COVID-19 susceptibility in pregnancy: Immune/inflammatory considerations, the role of placental ACE-2 and research considerations. Reprod Biol (2020) 20(4):568–72. doi: 10.1016/j.repbio.2020.10.005 PMC7832785 33183974

[46] Li M Chen L Zhang J Xiong C Li X. The SARS-CoV-2 receptor ACE2 expression of maternal-fetal interface and fetal organs by single-cell transcriptome study. PloS One (2020) 15(4):e0230295. doi: 10.1371/journal.pone.0230295 32298273 PMC7161957

[47] Singh M Bansal V Feschotte C. A single-cell RNA expression map of human coronavirus entry factors. Cell Rep (2020) 32(12):108175. doi: 10.1016/j.celrep.2020.108175 32946807 PMC7470764

[48] Dong L Pei S Ren Q Fu S Yu L Chen H. Evaluation of vertical transmission of SARS-CoV-2 *in utero*: Nine pregnant women and their newborns. Placenta (2021) 111:91–6. doi: 10.1016/j.placenta.2021.06.007 PMC8245148 34217121

[49] Babal P Krivosikova L Sarvaicova L Deckov I Szemes T Sedlackova T. Intrauterine fetal demise after uncomplicated COVID-19: What can we learn from the case? Viruses (2021) 13(12):2545. doi: 10.3390/v13122545 34960815 PMC8708385

[50] Huang Z Xia S Mei S Wen Y Liu J Dong C. Integrated analysis reveals the characteristics and effects of SARS-CoV-2 maternal-fetal transmission. Front Microbiol (2022) 13:813187. doi: 10.3389/fmicb.2022.813187 35154056 PMC8828581

[51] Mourad M Jacob T Sadovsky E Bejerano S Simone GS Bagalkot TR. Placental response to maternal SARS-CoV-2 infection. Sci Rep (2021) 11(1):14390. doi: 10.1038/s41598-021-93931-0 34257394 PMC8277865

[52] Tallarek AC Urbschat C Fonseca Brito L Stanelle-Bertram S Krasemann S Frascaroli G. Inefficient placental virus replication and absence of neonatal cell-specific immunity upon sars-CoV-2 infection during pregnancy. Front Immunol (2021) 12:698578. doi: 10.3389/fimmu.2021.698578 34149740 PMC8211452

[53] Argueta LB Lacko LA Bram Y Tada T Carrau L Rendeiro AF. Inflammatory responses in the placenta upon SARS-CoV-2 infection late in pregnancy. iScience (2022) 25(5):104223. doi: 10.1016/j.isci.2022.104223 35434541 PMC8996470

[54] Liu F Han K Blair R Kenst K Qin Z Upcin B. SARS-CoV-2 infects endothelial cells *In vivo* and *In vitro* . Front Cell Infect Microbiol (2021) 11:701278. doi: 10.3389/fcimb.2021.701278 34307198 PMC8292147

[55] Schimmel L Chew KY Stocks CJ Yordanov TE Essebier P Kulasinghe A. Endothelial cells are not productively infected by SARS-CoV-2. Clin Transl Immunol (2021) 10(10):e1350. doi: 10.1002/cti2.1350 PMC8542944 34721846

[56] Rajah MM Bernier A Buchrieser J Schwartz O. The mechanism and consequences of SARS-CoV-2 spike-mediated fusion and syncytia formation. J Mol Biol (2022) 434(6):167280. doi: 10.1016/j.jmb.2021.167280 34606831 PMC8485708

[57] Buchrieser J Degrelle SA Couderc T Nevers Q Disson O Manet C. IFITM proteins inhibit placental syncytiotrophoblast formation and promote fetal demise. Science (2019) 365(6449):176–80. doi: 10.1126/science.aaw7733 31296770

[58] Juttukonda LJ Wachman EM Boateng J Jain M Benarroch Y Taglauer ES. Decidual immune response following COVID-19 during pregnancy varies by timing of maternal SARS-CoV-2 infection. J Reprod Immunol (2022) 151:103501. doi: 10.1016/j.jri.2022.103501 35231754 PMC8867981

[59] Libby P Luscher T. COVID-19 is, in the end, an endothelial disease. Eur Heart J (2020) 41(32):3038–44. doi: 10.1093/eurheartj/ehaa623 PMC7470753 32882706

[60] Huertas A Montani D Savale L Pichon J Tu L Parent F. Endothelial cell dysfunction: a major player in SARS-CoV-2 infection (COVID-19)? Eur Respir J (2020) 56(1):2001634. doi: 10.1183/13993003.01634-2020 32554538 PMC7301835

[61] Gupta A Madhavan MV Sehgal K Nair N Mahajan S Sehrawat TS. Extrapulmonary manifestations of COVID-19. Nat Med (2020) 26(7):1017–32. doi: 10.1038/s41591-020-0968-3 PMC11972613 32651579

