# Supplementary figures and images for: SARS-CoV-2 modulates virus receptor expression in placenta and can induce trophoblast fusion, inflammation and endothelial permeability
Source: Front Immunol. 2022 Sep 13;13:957224. doi: 10.3389/fimmu.2022.957224 (PMC9513489; doi:10.3389/fimmu.2022.957224)

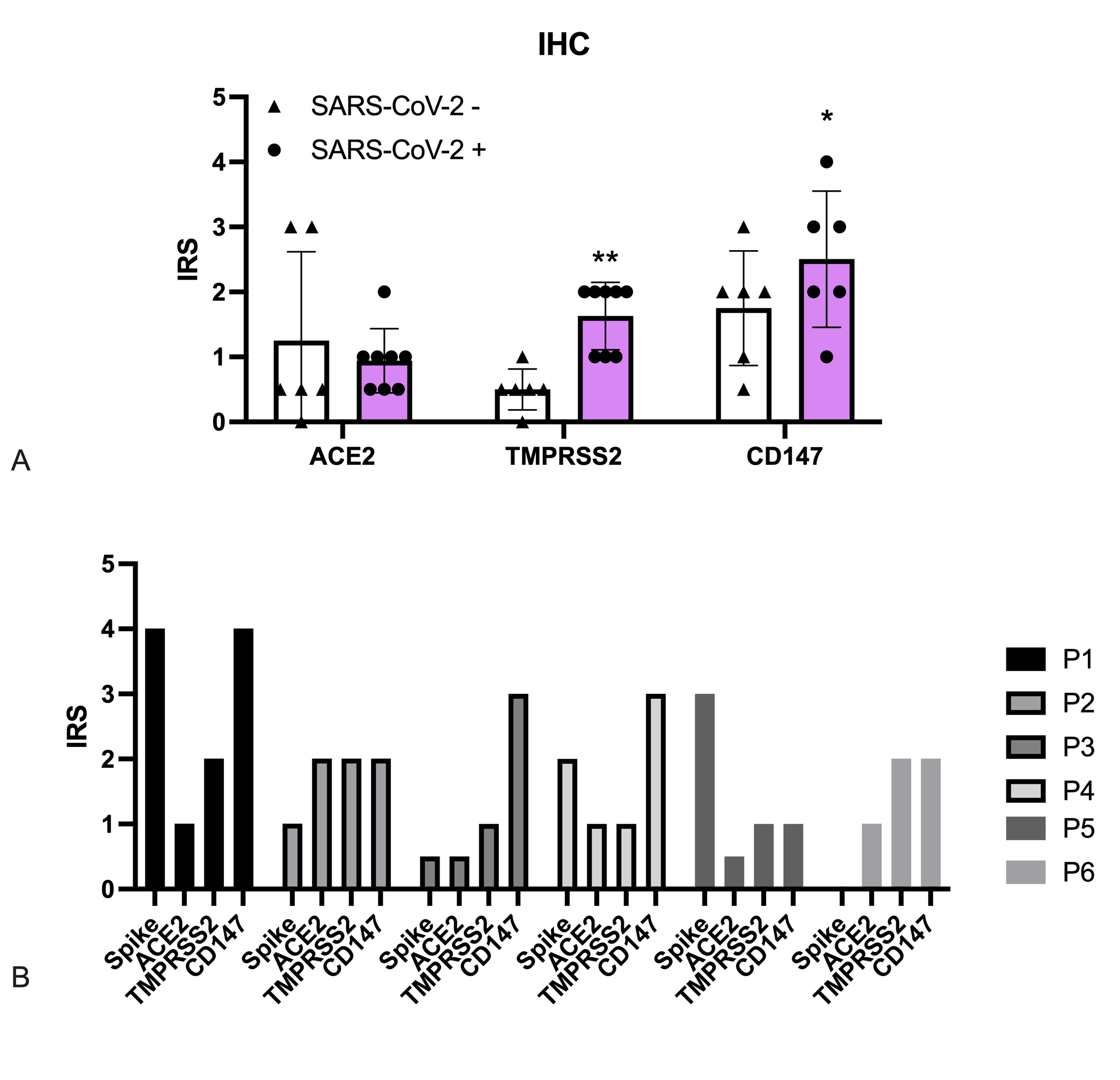

Supplement: Supplementary file 1 [file Image_1.jpeg]

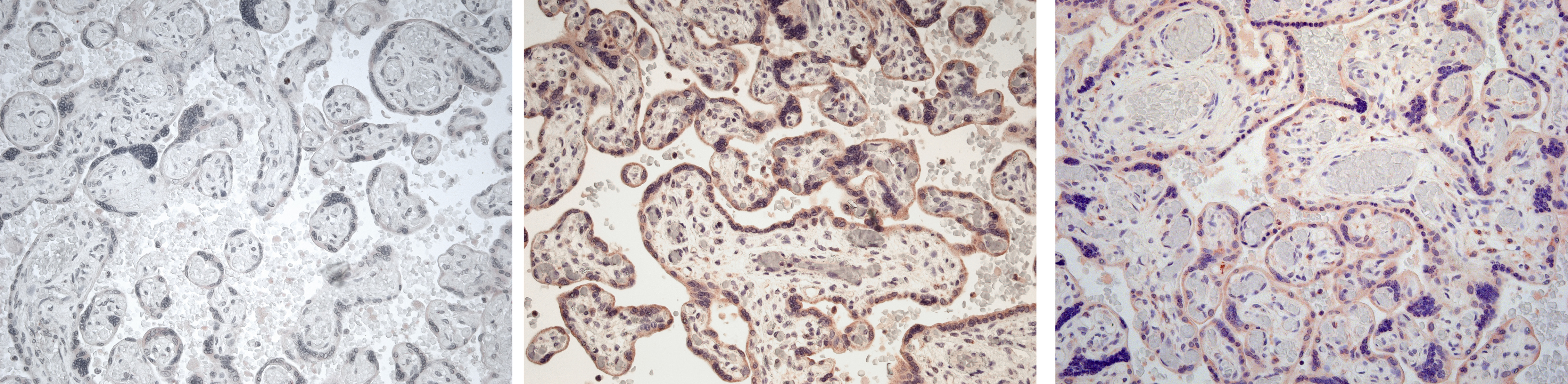

Supplement: Supplementary file 2 [file Image_2.jpeg]

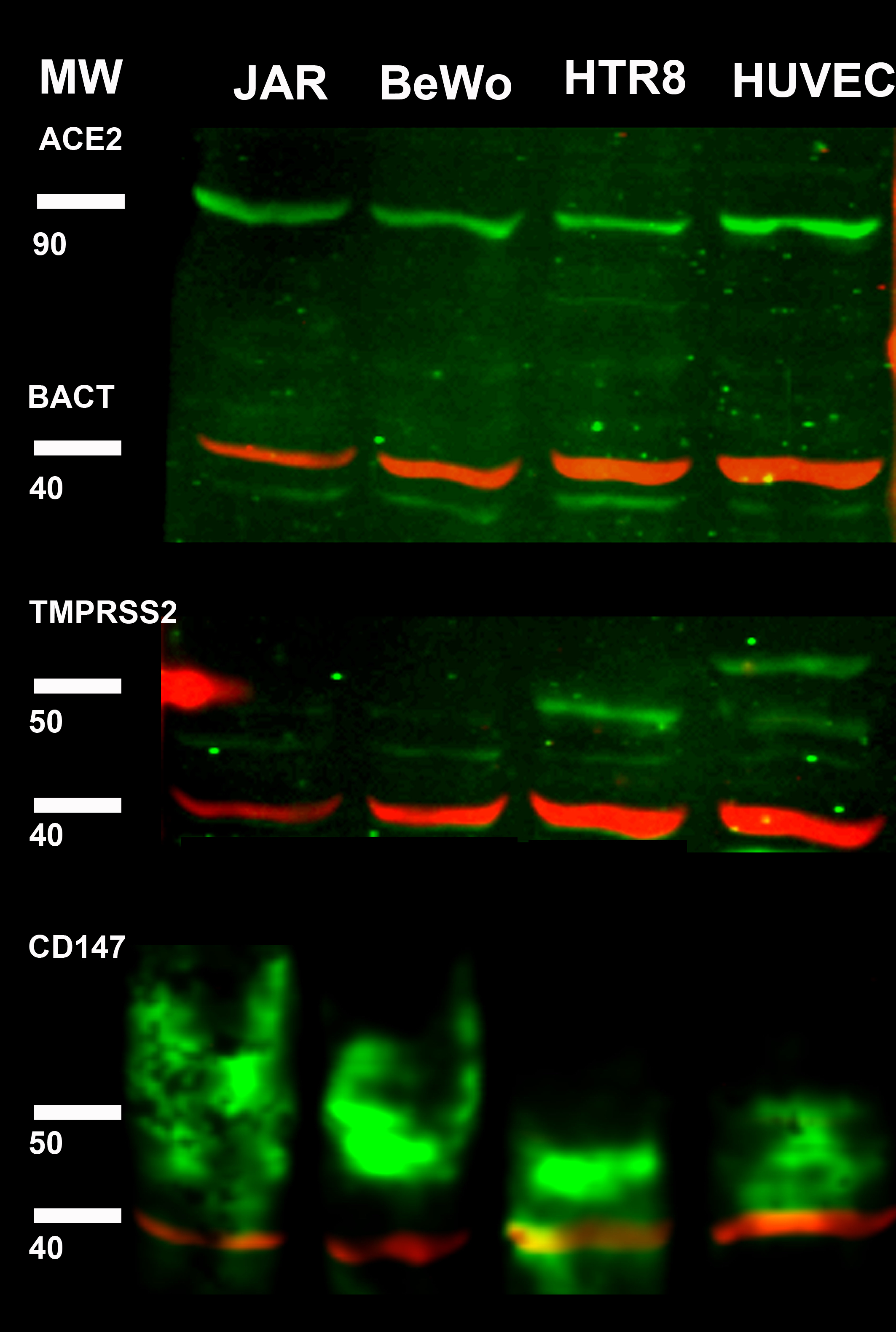

Supplement: Supplementary file 3 [file Image_3.jpeg]

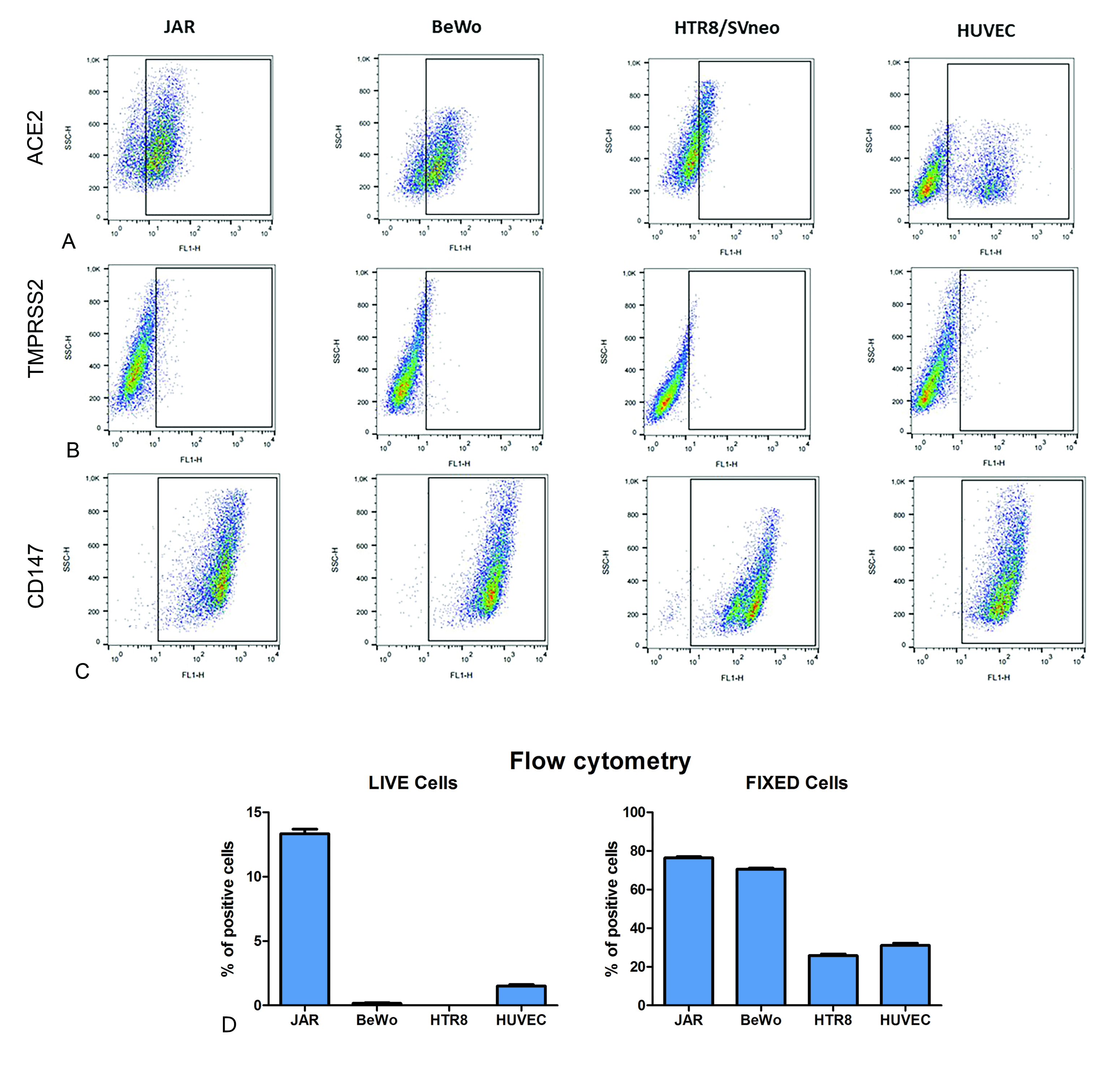

Supplement: Supplementary file 4 [file Image_4.jpeg]

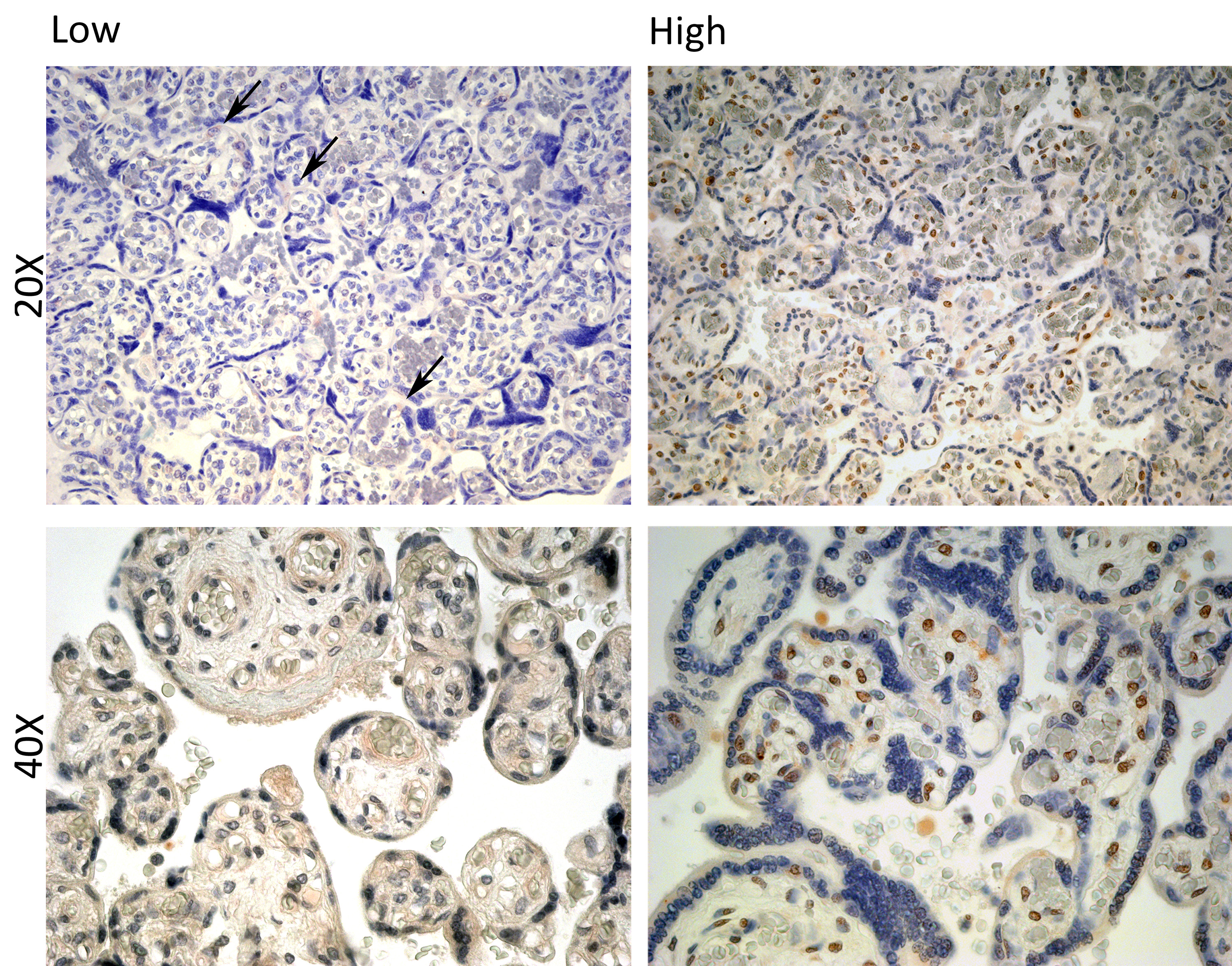

Supplement: Supplementary file 5 [file Image_5.jpeg]

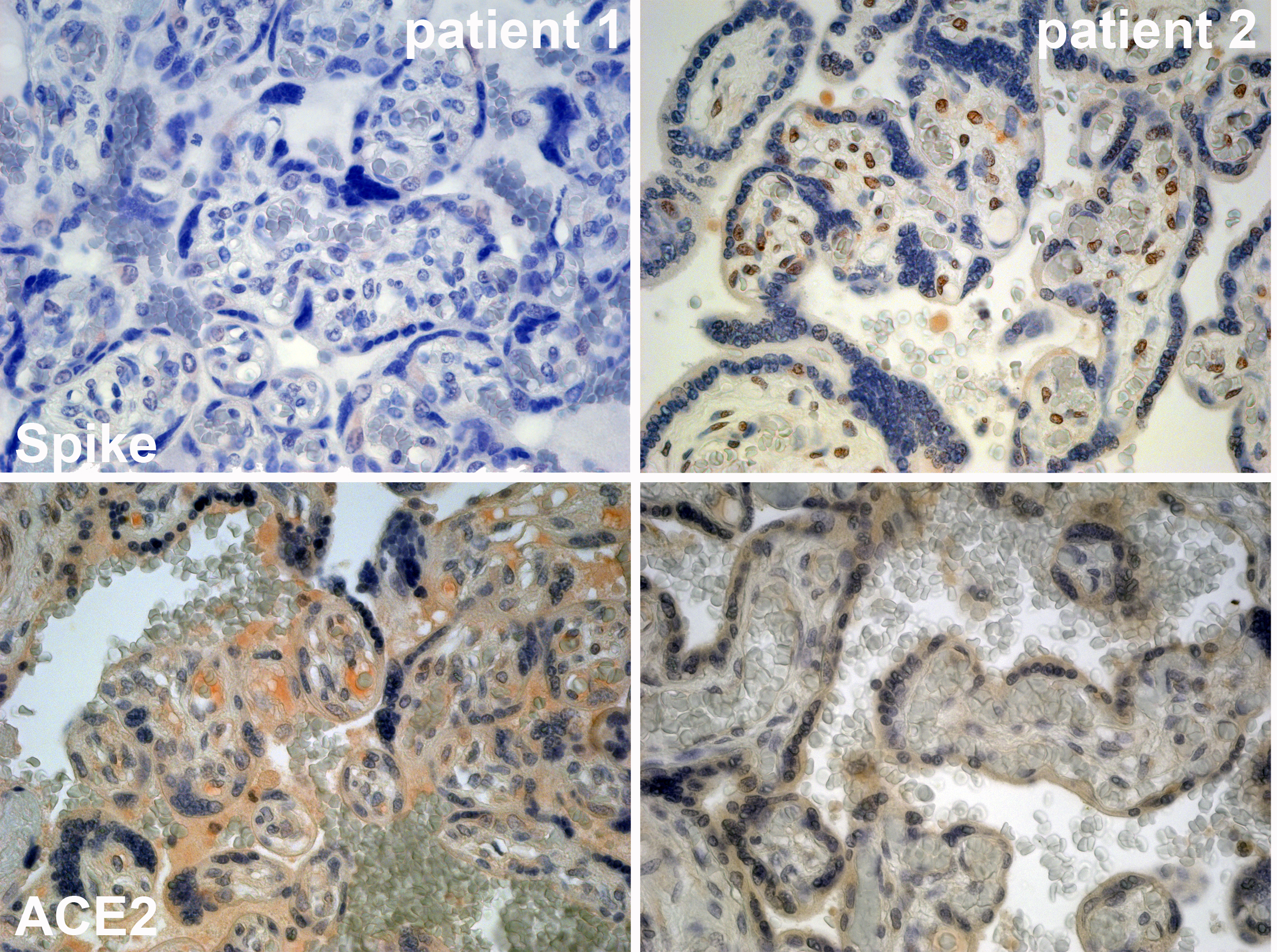

Supplement: Supplementary file 6 [file Image_6.jpeg]

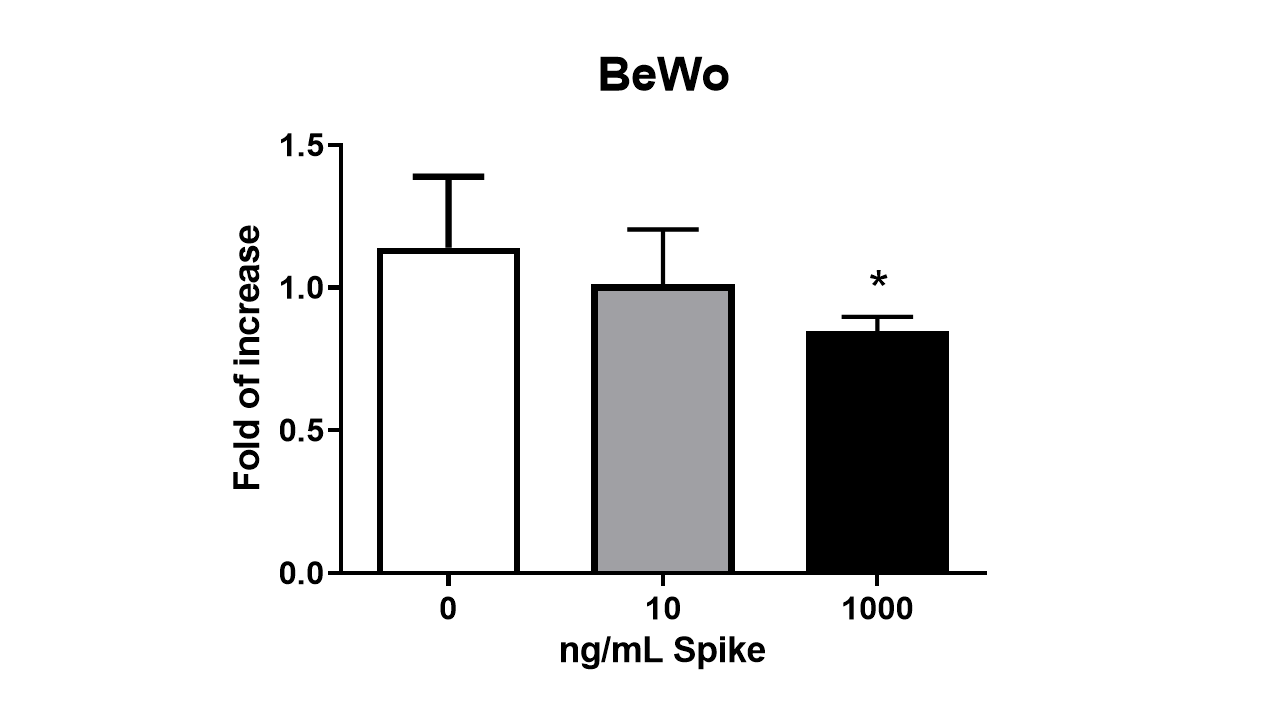

Supplement: Supplementary file 7 [file Image_7.tif]

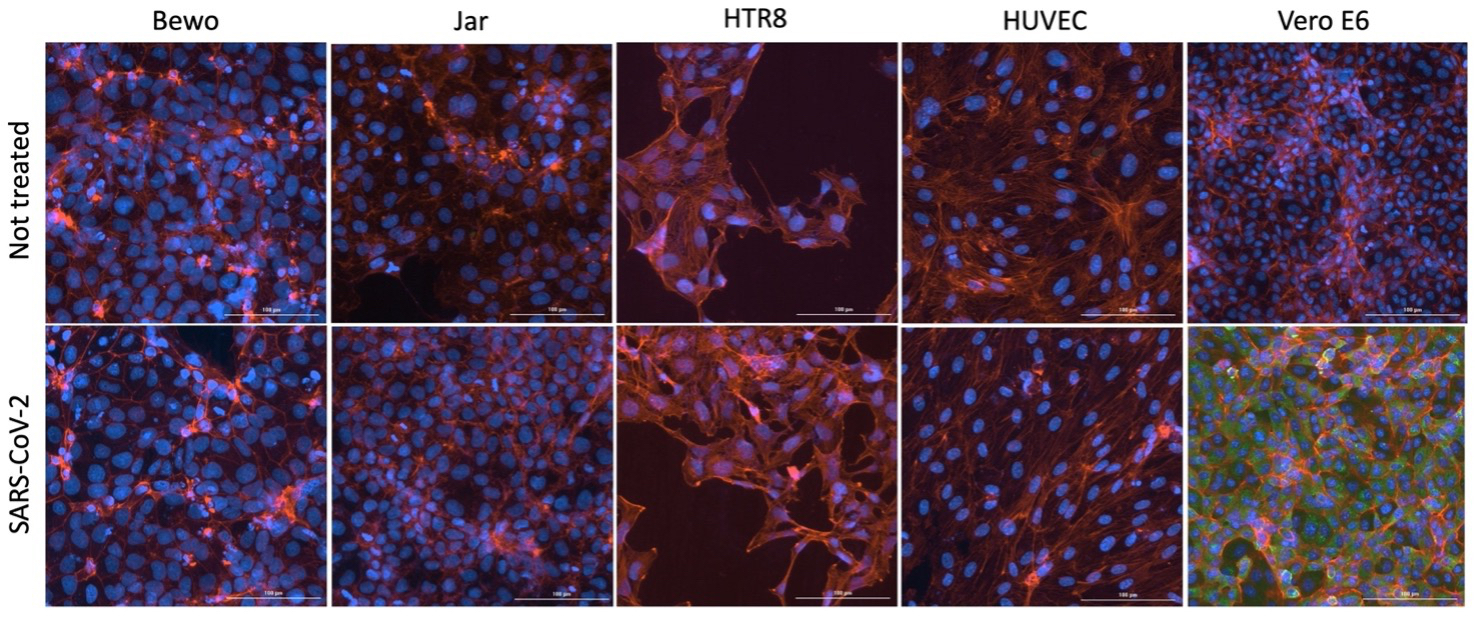

Supplement: Supplementary file 8 [file Image_8.jpeg]

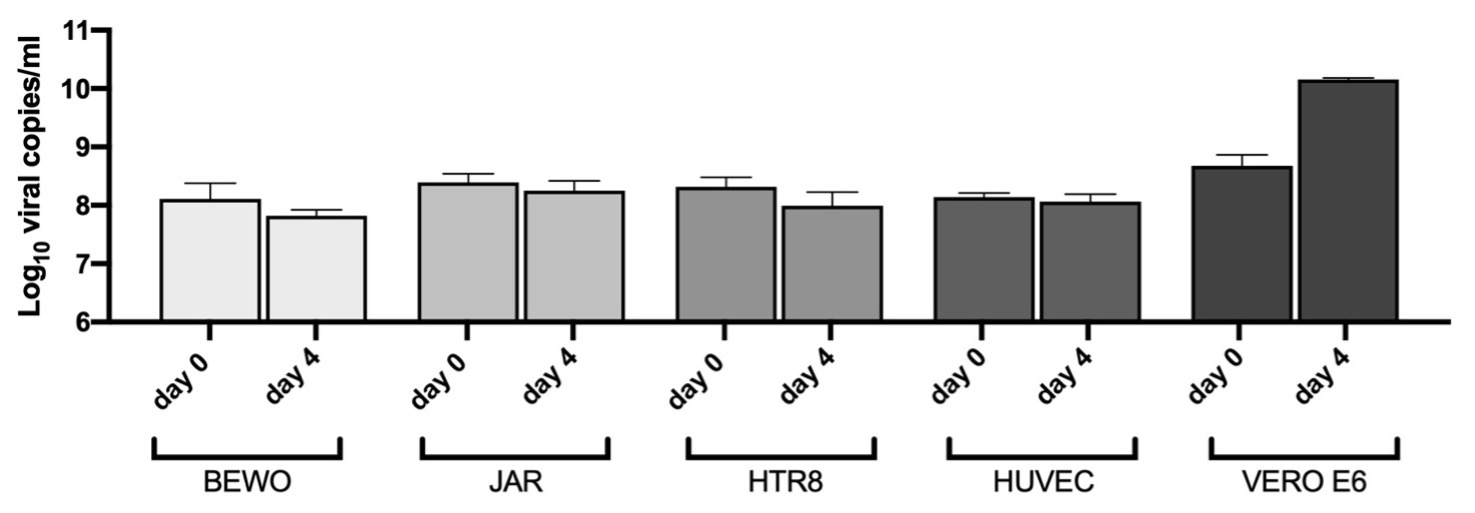

Supplement: Supplementary file 9 [file Image_9.jpeg]

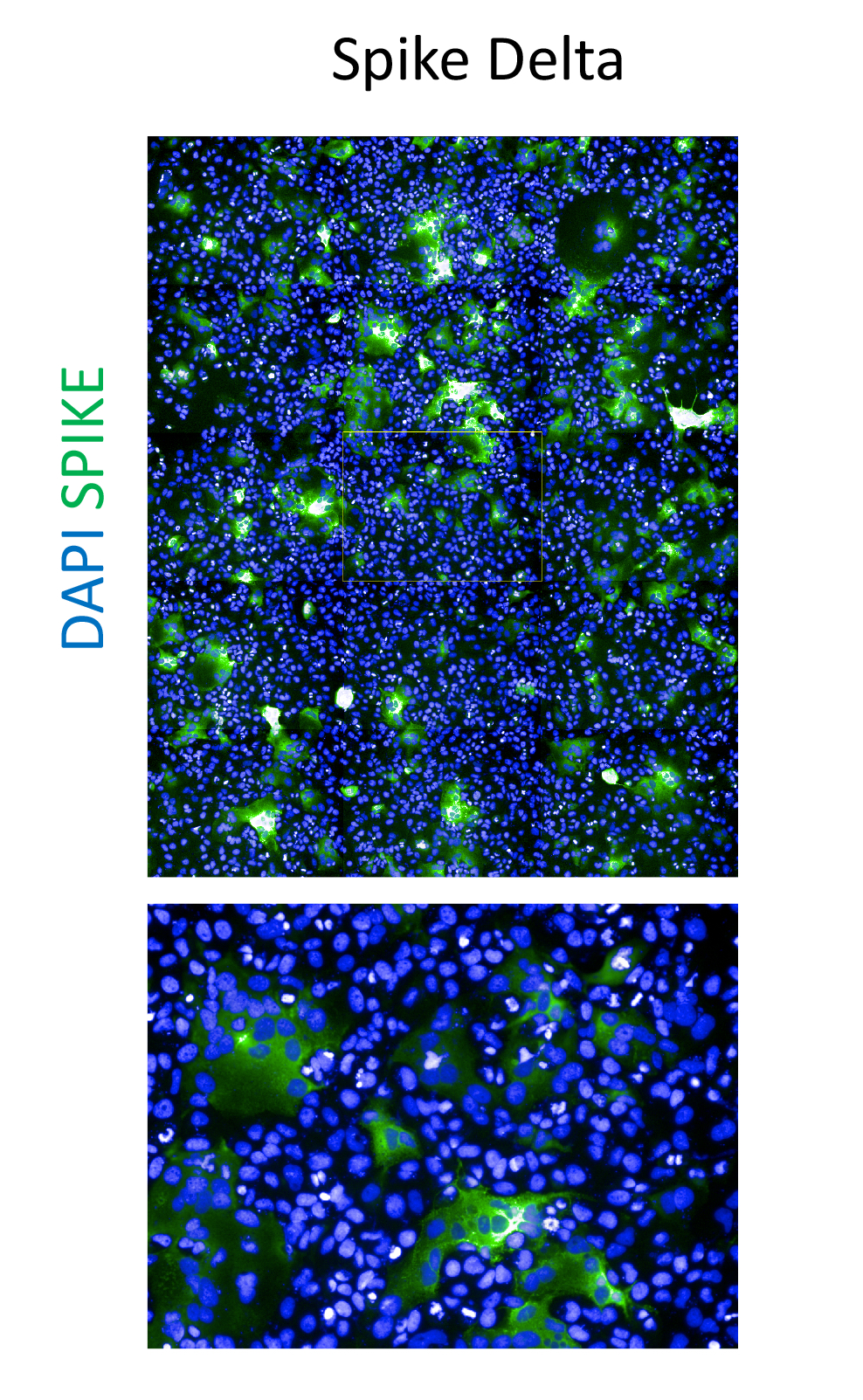

Supplement: Supplementary file 10 [file Image_10.jpeg]

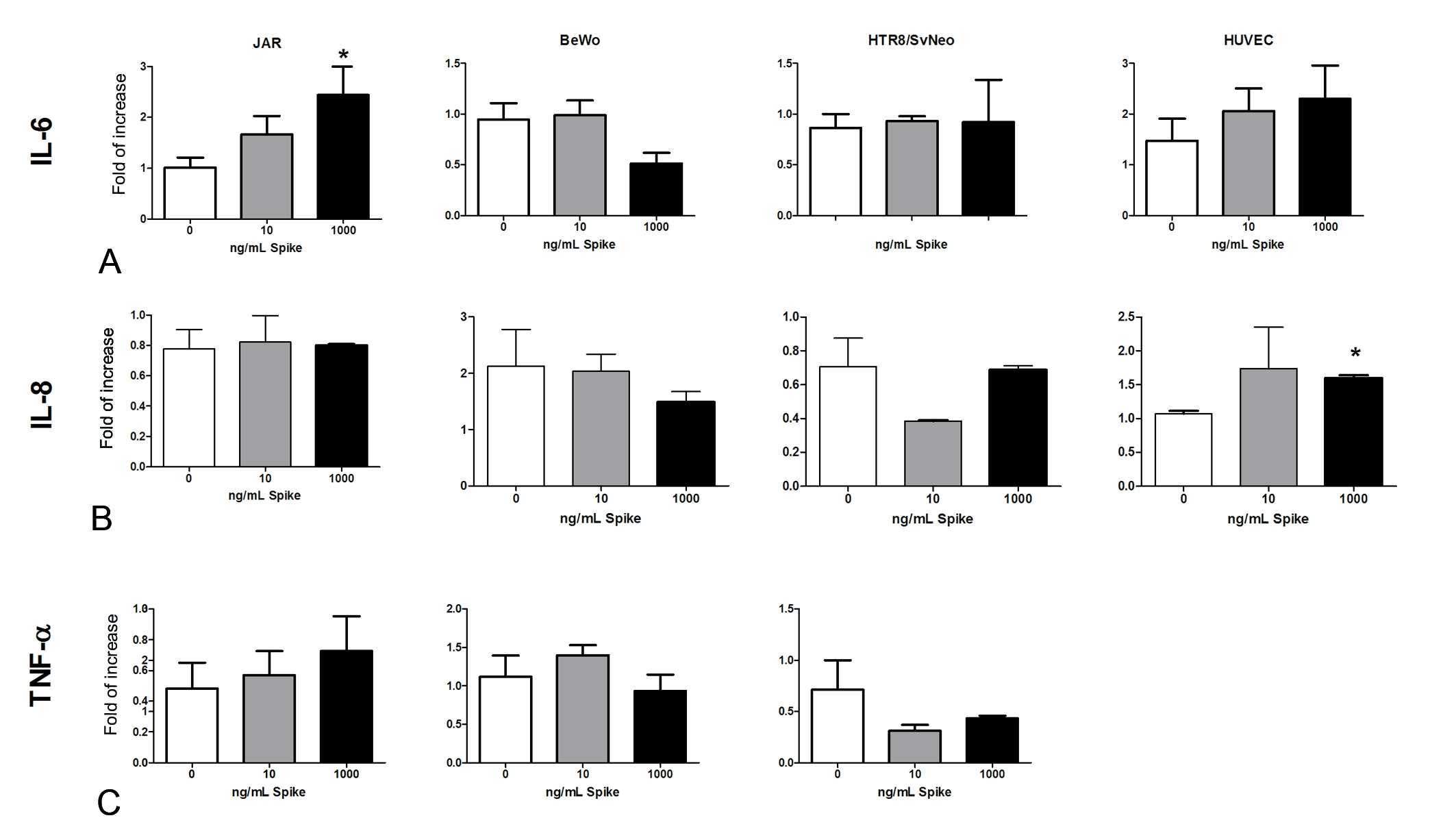

Supplement: Supplementary file 11 [file Image_11.tif]
