# Supplementary material for: SARS-CoV-2 modulates virus receptor expression in placenta and can induce trophoblast fusion, inflammation and endothelial permeability
Source: Front Immunol. 2022 Sep 13;13:957224. doi: 10.3389/fimmu.2022.957224 (PMC9513489; doi:10.3389/fimmu.2022.957224)
Supplement: Supplementary file 12 [file DataSheet_1.docx]

***Supplementary Material***

**
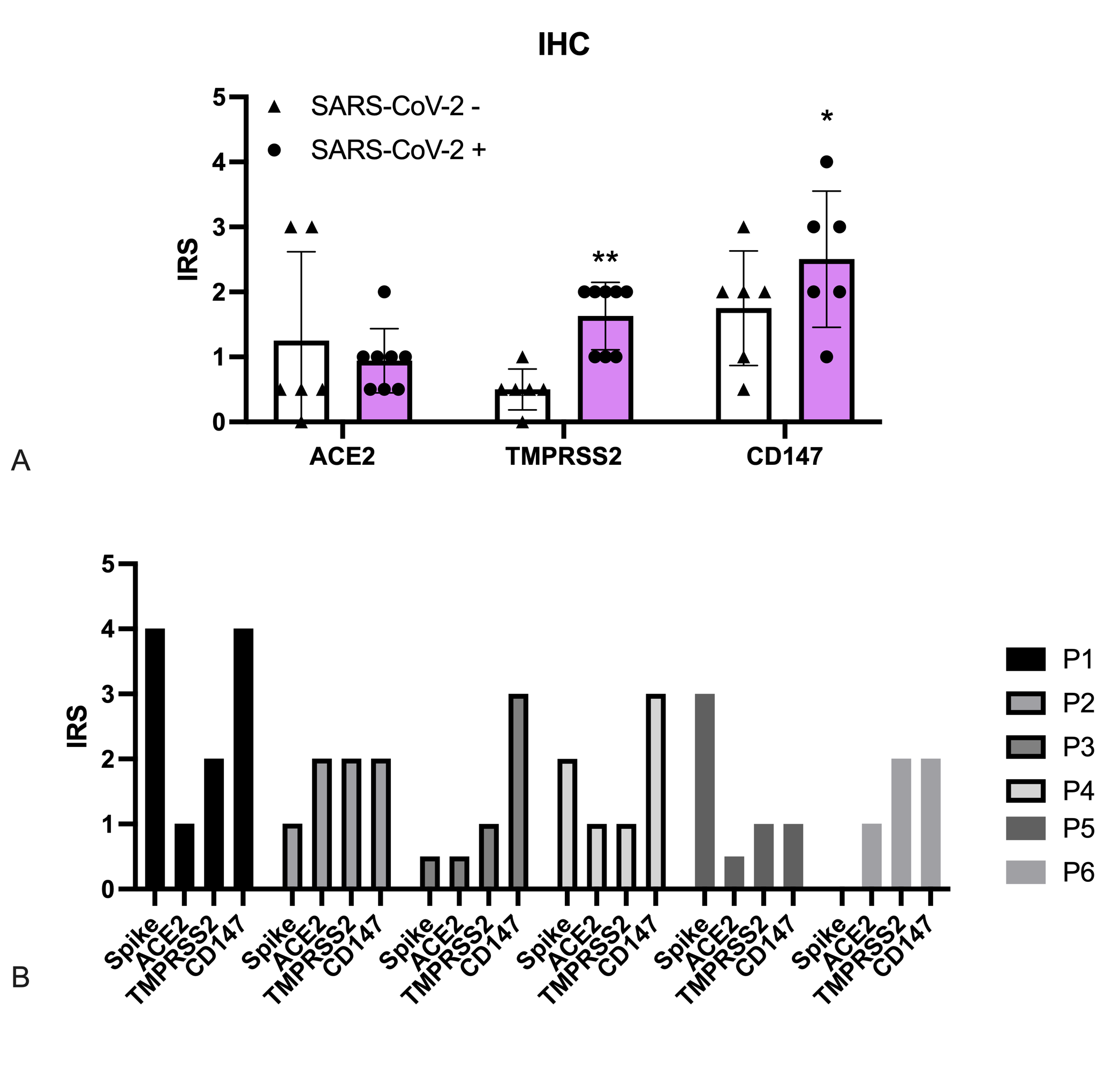
**

**Supplementary Figure 1.** Quantification of immunohistochemical analysis for ACE2, TMPRSS2 and CD147 in SARS-CoV-2- and SARS-CoV-2+ placentae. (**A**) To quantify the expression of entry receptors in placentae, we utilized an immunoreactive score (IRS). For each slide, we analyzed three different visual fields of the microscope, attributing a score of positivity ranging from 0 to 4. (**B**) Histograms representative of Spike protein presence compared to ACE2, TMPRSS2 and CD147 expression in six different SARS-CoV-2+ placentae, expressed as IRS**.**

**
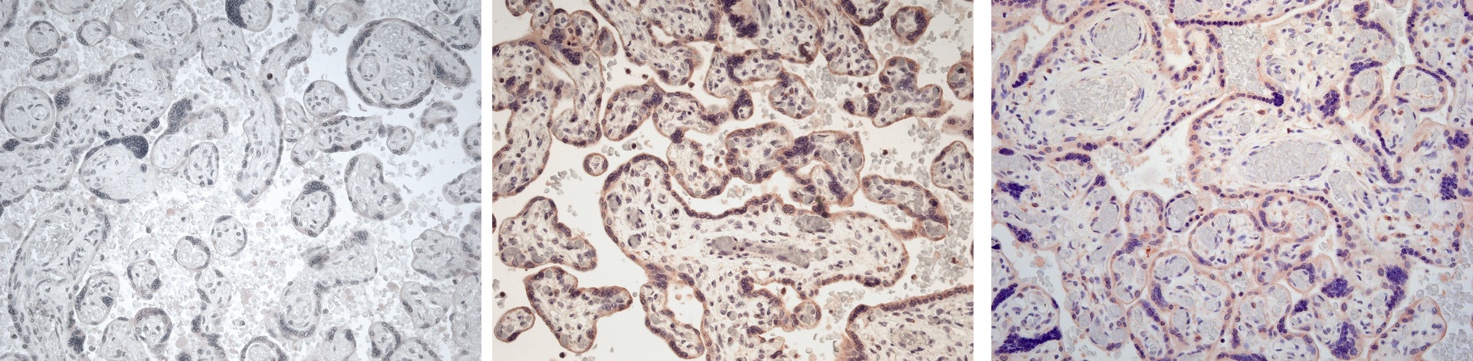
**

**Supplementary Figure 2**. Immunohistochemical staining of healthy placentae for ACE2. ACE2 positivity showed a great variability among patients. Streptavidin–biotin–peroxidase complex system with AEC (red) chromogen.


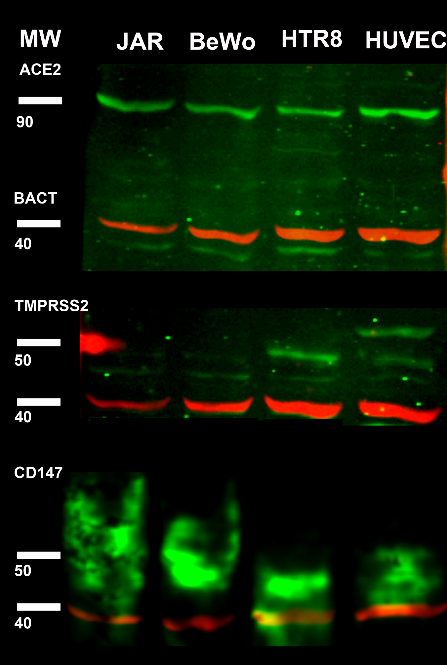


**Supplementary Figure 3**. Representative image of Western blot analyses of SARS-CoV-2 receptors in placental cell lines. Cell lysates of JAR, BeWo, HTR8 and HUVEC were separated by SDS-PAGE. After transfer, membrane was probed with α-ACE2, α-TMPRSS2 and α-CD147 primary antibodies and then with IRDye 800CW secondary antibody. β-actin was used to normalize the results. Signal intensity was detected using an Odyssey CLx near-infrared scanner (LI-COR Biosciences, Lincoln, NE, USA). Image acquisition and processing were performed with Image Studio 5.2 (LI-COR Biosciences).

**
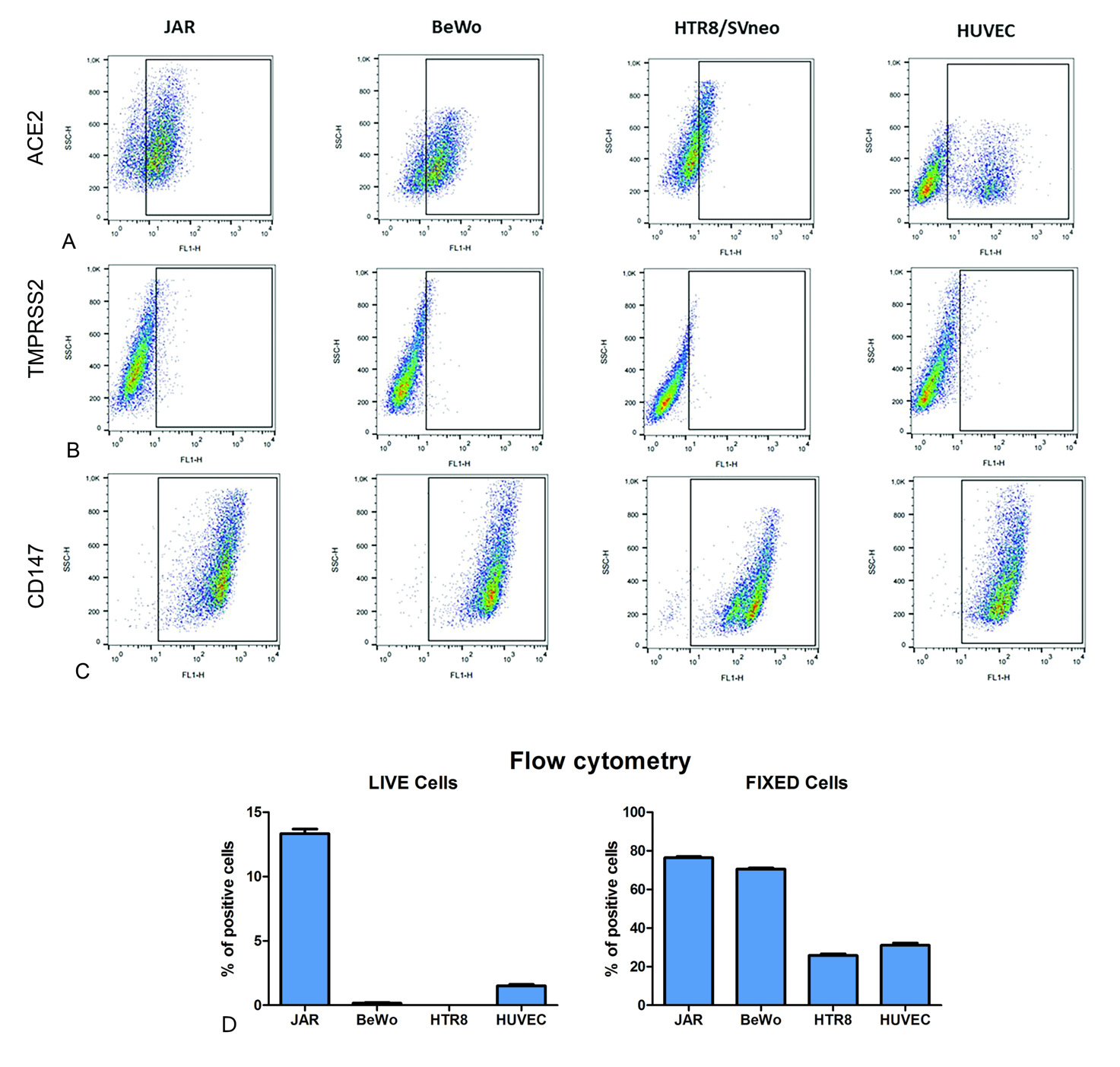
**

**Supplementary Figure 4**. Flow cytometry characterization of ACE2 (**A**), TMPRSS2 (**B**) and CD147 (**C**) in live placental cells. (**D**) Cytofluorimetric analyses for ACE2 were conducted on both live and fixed cells.


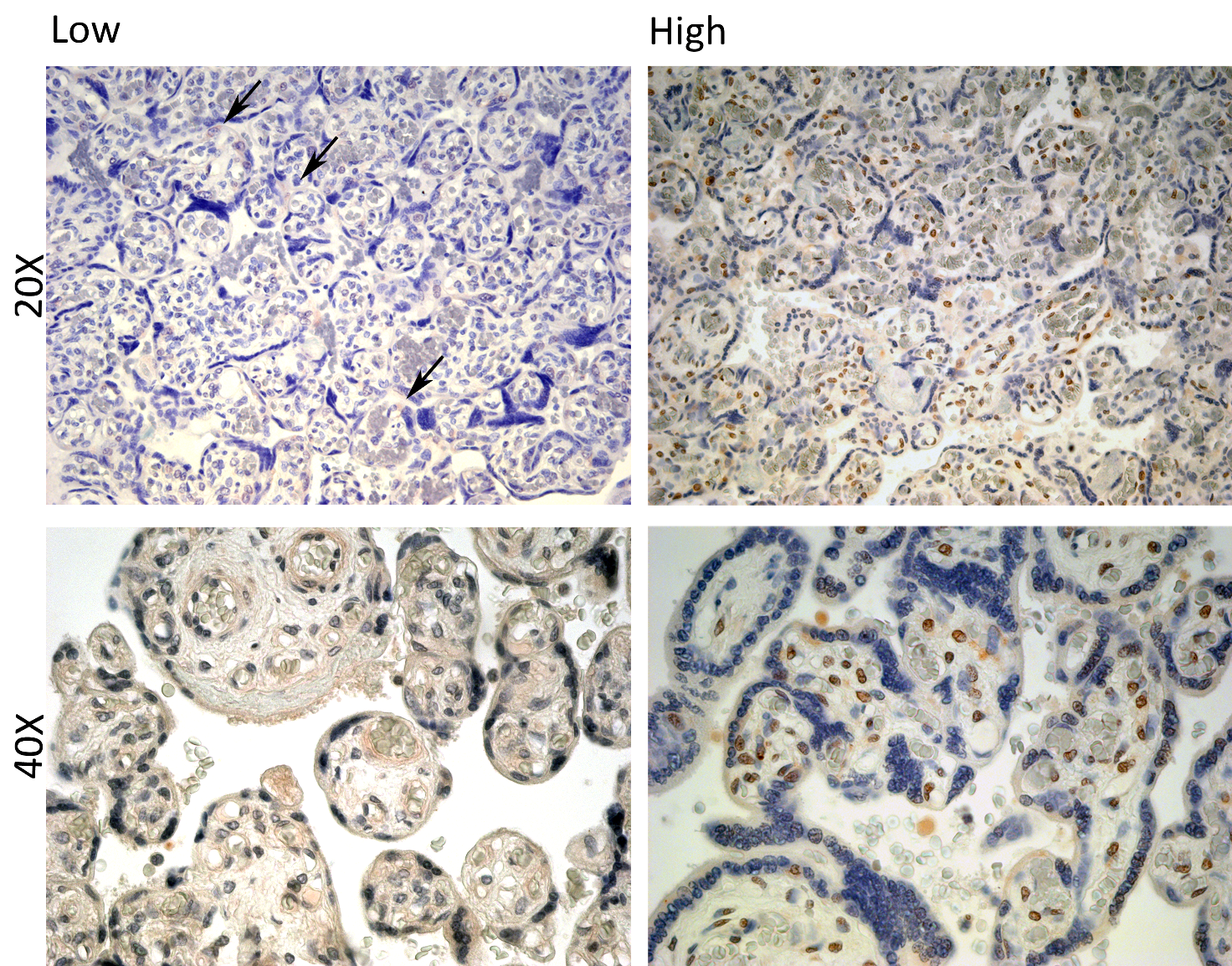


**Supplementary Figure 5**. Immunohistochemical analyses for Spike protein (S2) in infected placentae resulted in a variable detection of SARS-CoV-2 in the tissues (low presence vs. high presence). Streptavidin–biotin–peroxidase complex system with AEC (red) chromogen.


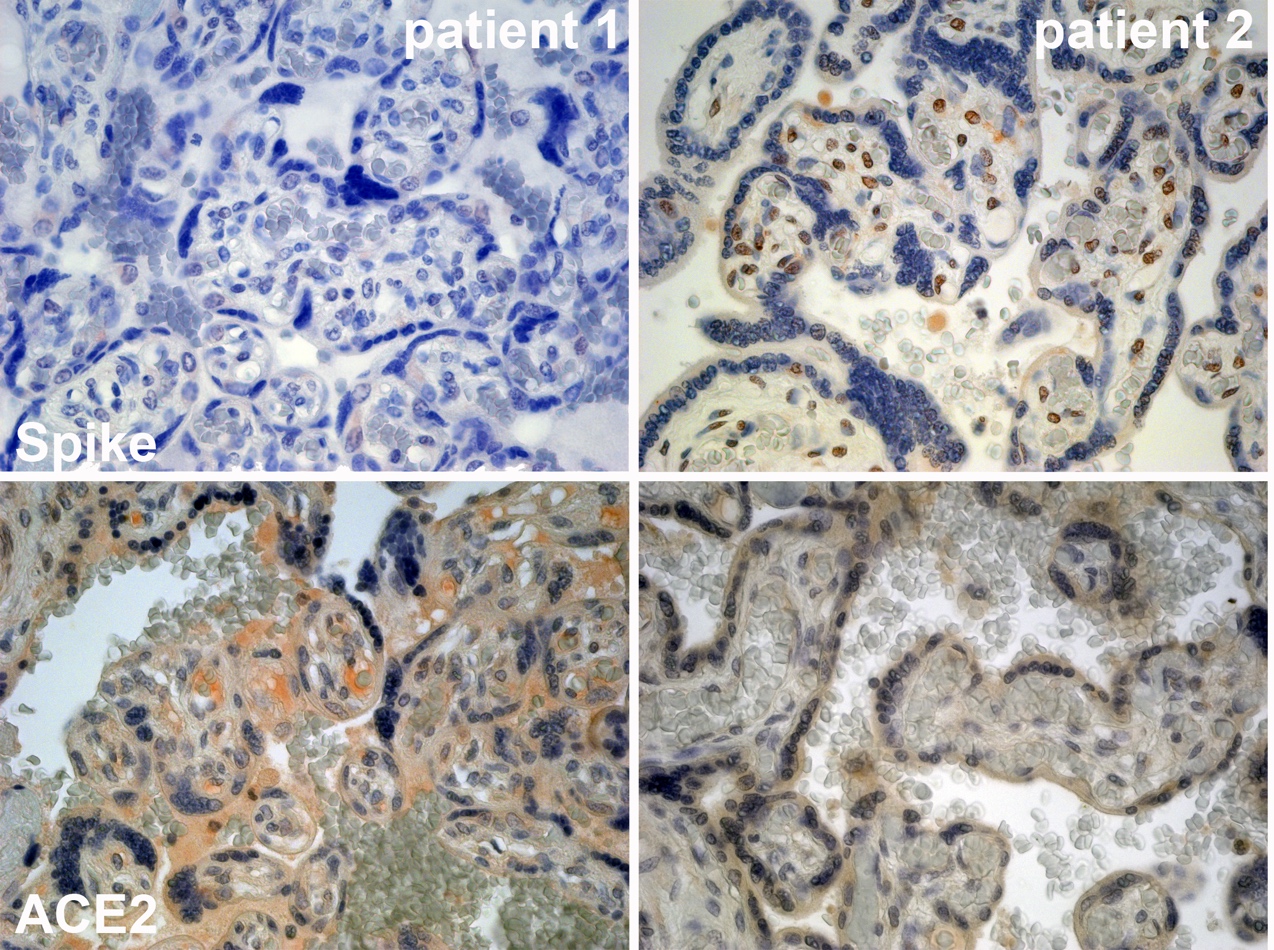


**Supplementary Figure 6**. IHC staining for Spike protein and for ACE2 onto infected placentae. Patient 1 displayed a low expression of Spike protein, but a high expression of its receptor ACE2. Patient 2 conversely had a high expression of Spike protein, but a lower manifestation of ACE2.


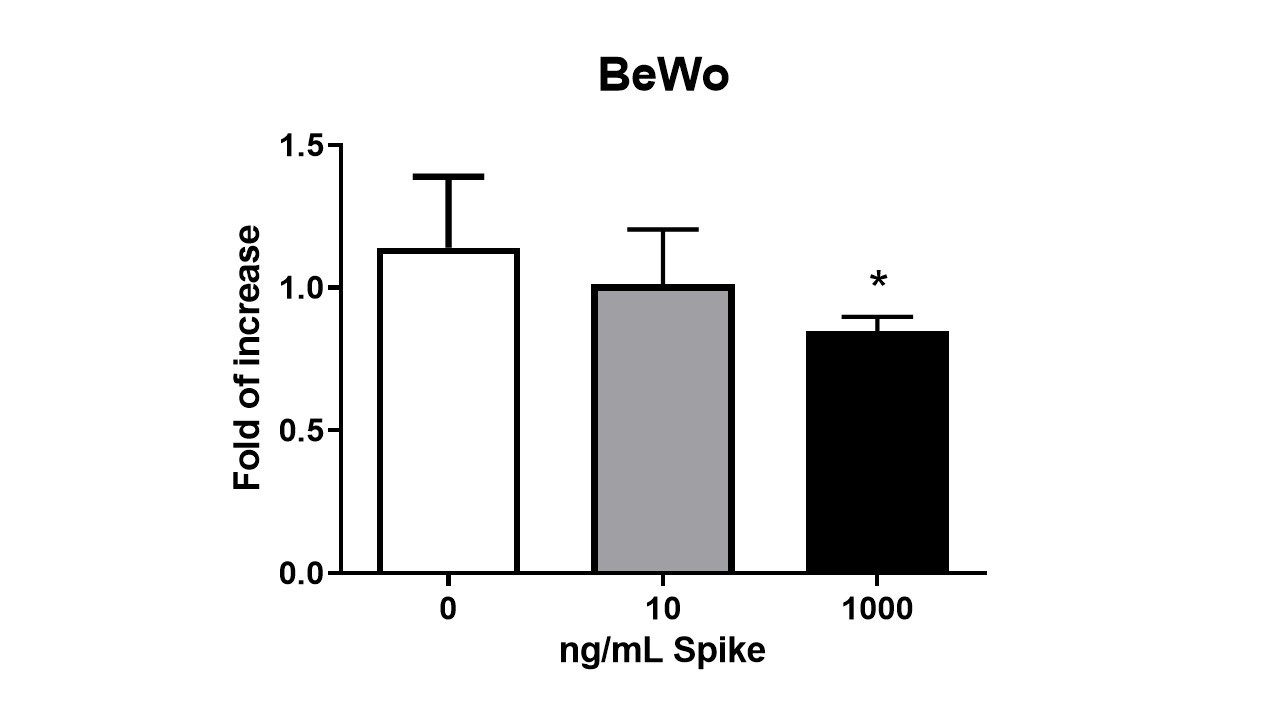


**Supplementary Figure 7**. Results of RT-qPCR for *ACE2* mRNA expression in Spike-stimulated BeWo. Graph shows a downregulation of *ACE2* expression by stimulated cells, with a significative effect at 1000 ng/mL of Spike protein. **p* < 0.001 with respect to untreated cells (Mann-Whitney test).


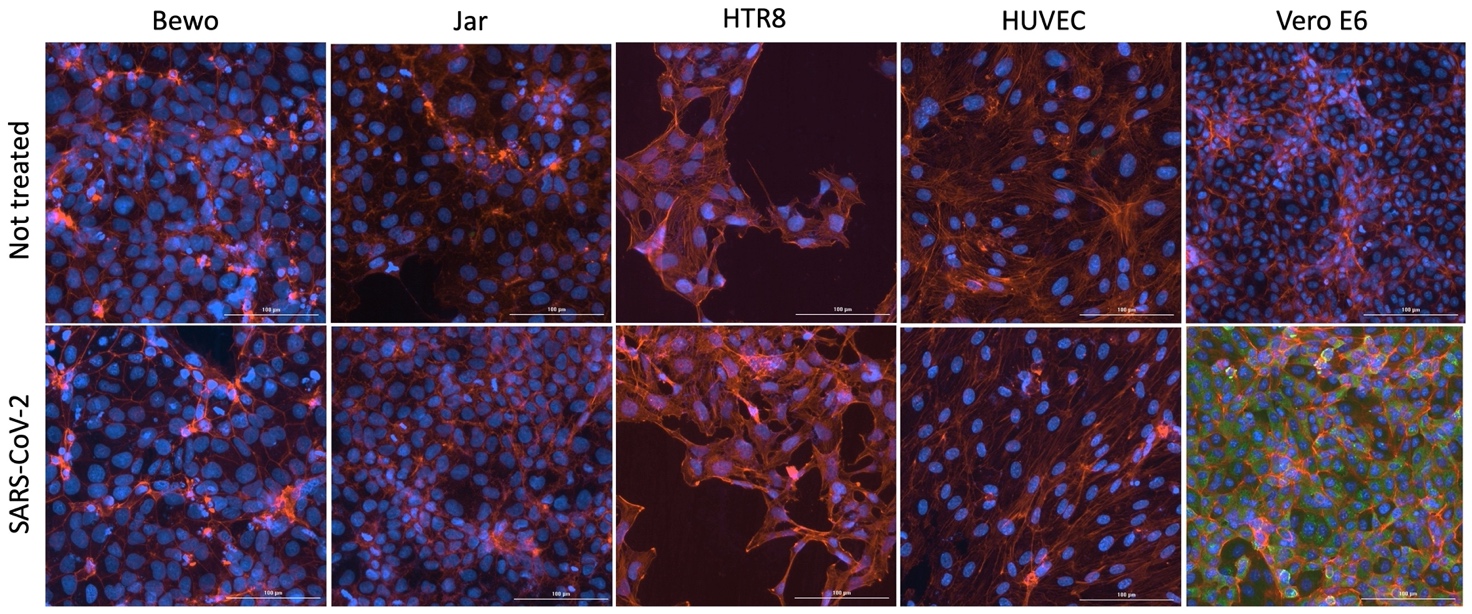


**Supplementary Figure 8**. Immunofluorescence staining of nucleocapsid viral protein in BeWo, JAR, HTR-8/SVneo, HUVEC and Vero E6 cells (nucleus in blue, nucleocapsid in green, actin in red). Untreated cells and cells infected with SARS-CoV-2 are displayed. Images were taken with Cytation 5 Cell Imaging Multi-Mode Reader (Biotek, Winooski, VT, USA). Scale bar 100 μM.


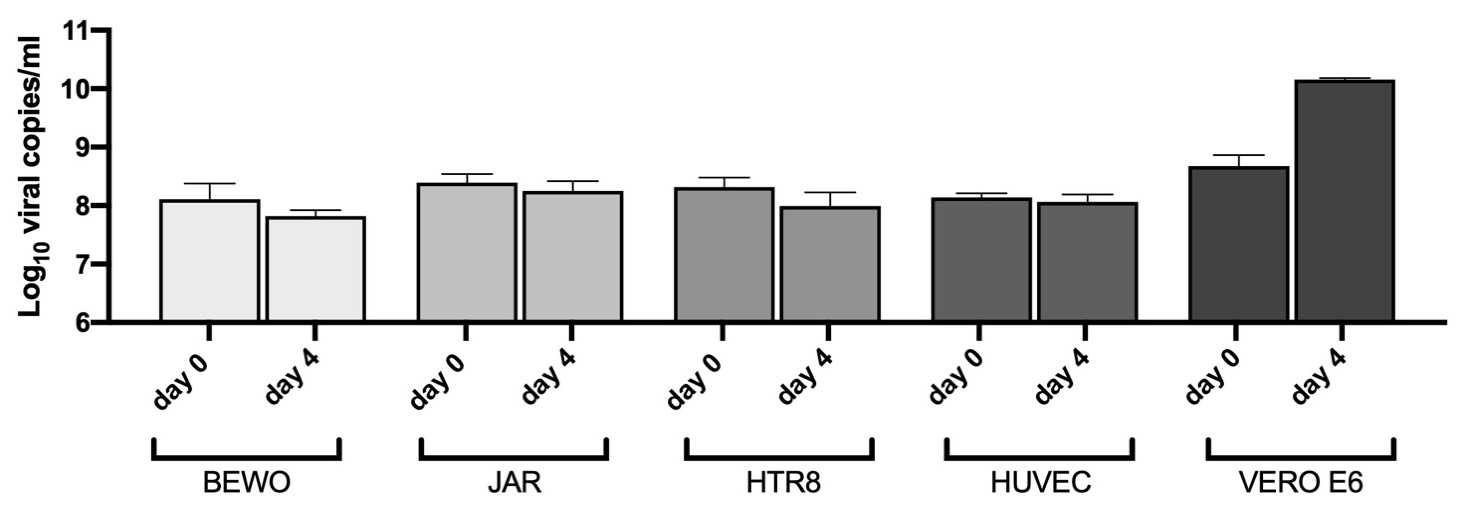


**Supplementary Figure 9**. Quantification by RT-qPCR of SARS-CoV-2 RNA presence in the supernatant of cell lines (Bewo, Jar, HTR8/SVneo, HUVEC, and Vero E6) incubated with the virus. The cells were challenged with SARS-CoV-2 for 24 hours, then incubated for 1 hour with fresh virus. Vero E6 cells were used as a control. The viral load was displayed as Log10 viral copies/ml (mean ± SD) at day 0, 4 and 7 post infection.


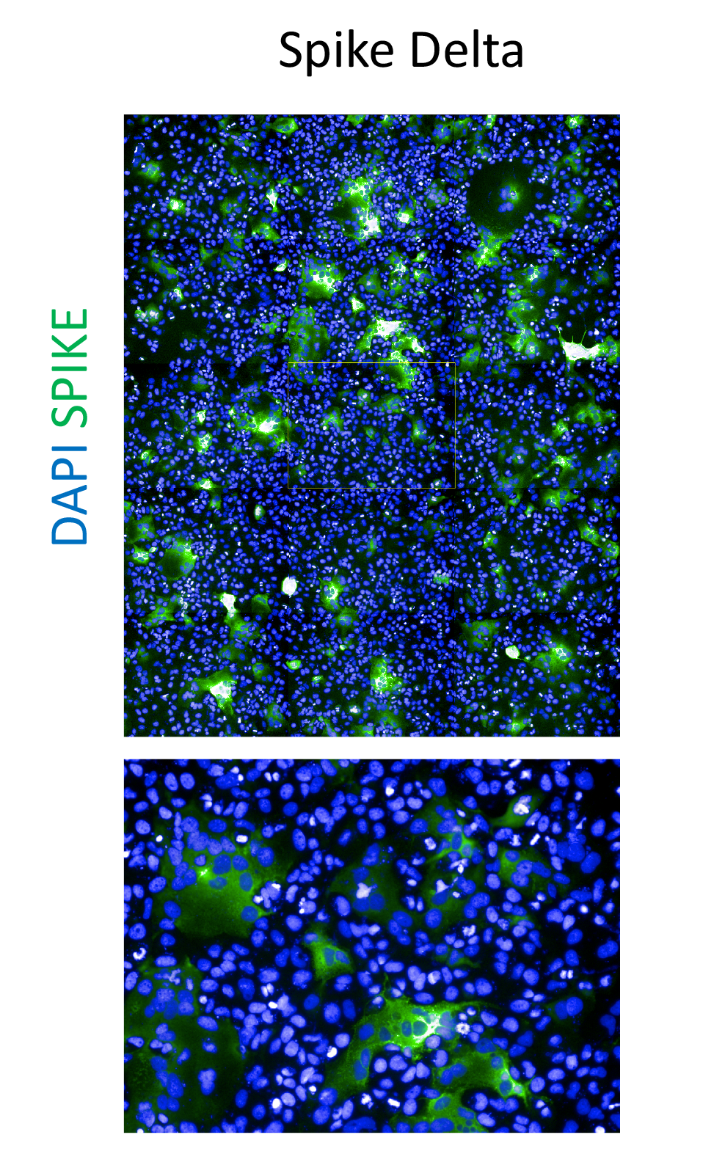


**Supplementary Figure 10.** Cell Fusion Assay. Vero E6 cells were seeded 10h before transfection. Cells were then transfected with pCMV-SPIKEDelta-V5+PCMV-hACE2, pCMV-SPIKEDelta-V5+PcDNA3 or pCMV-GFP+PcDNA3. Representative images of green fluorescence protein (GFP) and immunostaining for SARS-CoV2-Spike in Vero E6 taken using the Operetta high content screening microscope (PerkinElmer) with Olympus 20 x (NA-0.45) objective.

**
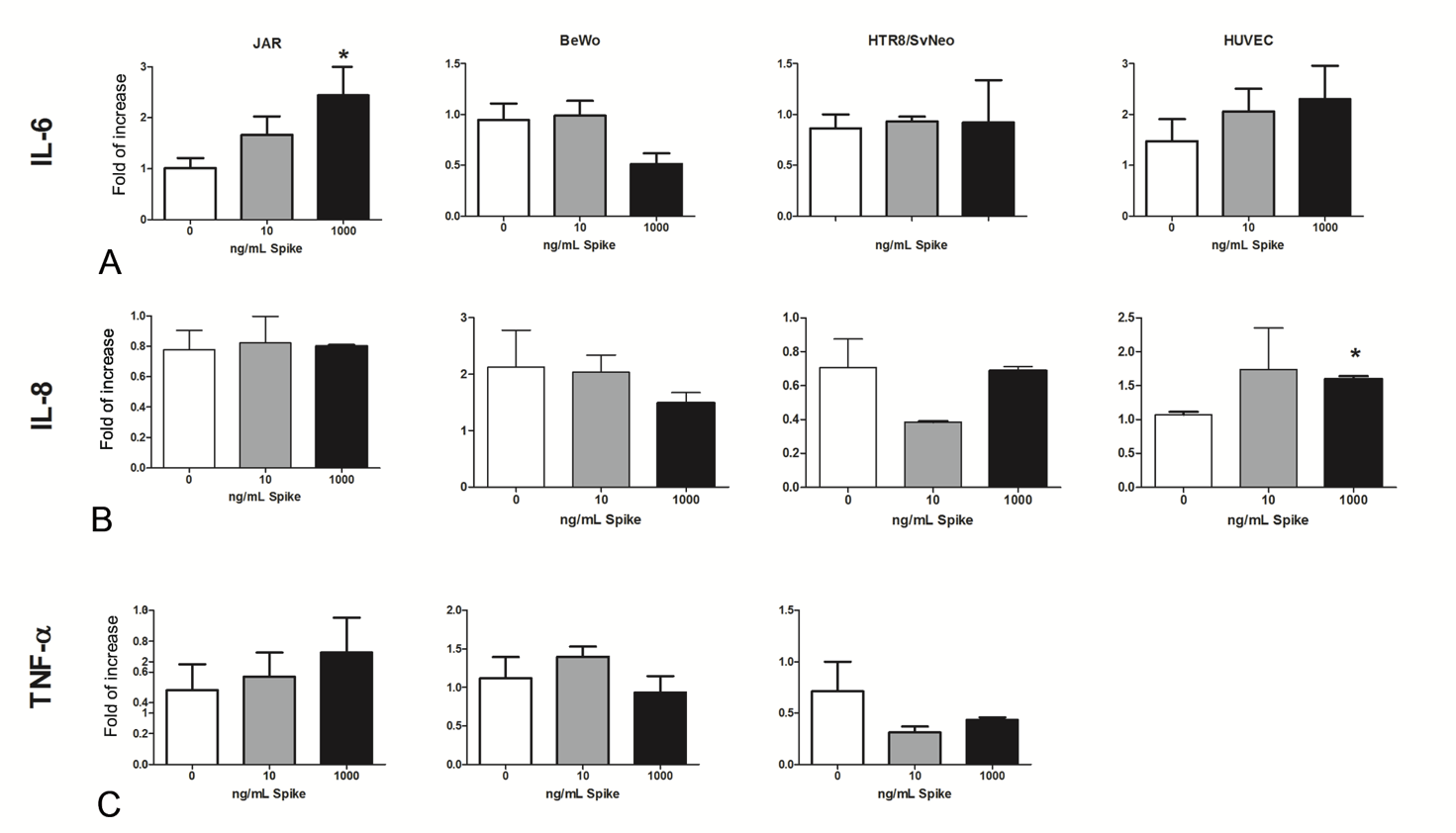
Supplementary Figure 11.** Pro-inflammatory effect of Spike protein on placental cells. RT-qPCR expression analysis of IL-6 (**A**), IL-8 (**B**) and TNF-α (**C**) in Spike-stimulated placental cell line. After 24h of treatment with 10 ng/mL or 1000 ng/mL of S1 Spike protein, total mRNA of JAR, BeWo, HTR8/SVneo or HUVEC was isolated and gene expression analysis was performed by RT-qPCR. The expression was normalized to the housekeeping genes *18S, ACTB* and *GAPDH*; results were mediated (geometric mean) and expressed as *fold of increase*. Data are expressed as mean ± SD of two independent experiments performed in duplicate. *p < 0.05
